# S-nitrosothiols loaded mini-sized Au@silica nanorod elicits collagen depletion and mitochondrial damage in solid tumor treatment

**DOI:** 10.7150/thno.42661

**Published:** 2020-05-20

**Authors:** Ping Liu, Yidan Wang, Yang Liu, Fengping Tan, Jining Li, Nan Li

**Affiliations:** 1 Tianjin Key Laboratory of Drug Delivery & High-Efficiency, School of Pharmaceutical Science and Technology, Tianjin University, 300072, Tianjin, P. R. China.; 2 School of Precision Instrument and Opto-Electronics Engineering, Tianjin University, 300072, Tianjin, P. R. China.; 3 Department of Orthopedic Surgery, Tianjin Medical University General Hospital, 300052, Tianjin, P. R. China.

**Keywords:** Nitric oxide, nanosweeper, collagen depletion, thermal-sensitive, RNS, mitochondrial-targeted

## Abstract

To a large extent, the dense extracellular matrix (ECM), which tightly connects tumor cells to arm the tumor into an intractable fortress, significantly decreases the nanoparticles delivery efficacy and overall performance in cancer treatments. Therefore, it is necessary to transform the dense stroma of solid tumors to loose state, which could realize deep penetration of nanomedicine and enhance cancer treatment effects. Here, we fabricated a protein-free collagen nanosweeper, triphenylphosphonium bromide (TPP) coated and S-nitrosothiols loaded mini-sized Au@silica nanorod (Au@SiO_2_-SNO/PEG/TPP, GSNP-TPP), to clear the transport barriers of nanoparticles as well as elevate enhanced permeability and retention (EPR) effect, thus alleviating the diffusion resistance and realizing further penetration of nanoparticles.

**Methods:** By modifying the Au@silica with thermo-sensitive S-nitrosothiols, the carrier could release the nitric oxide (NO) due to the surface overheat as well as perform photothermal therapy (PTT) under near-infrared (NIR) laser irradiation. The level of collagen depletion was observed via western blotting and immunofluorescent staining. In addition, the dual-imaging and antitumor efficiency of GSNP-TPPs were evaluated with the HeLa tumor-bearing mouse model.

**Results:** On one hand, the released NO could deplete collagen by activating matrix metalloproteinases (MMPs) to break collagen fibers, thus loosening the dense ECM to enhance the cellular internalization. On the other hand, with the mitochondrial-targeted effect of TPP, the diffusible NO in tumor might rapidly interact with superoxide anion (O_2_Ÿ^-^) to produce highly toxic and powerful reactive nitrogen species (RNS) -- peroxynitrite (ONOO^-^), which resulted in mitochondrial damage to induce cell apoptosis. With the unique properties of mini-sized gold nanorods, the formulated nanoparticles exhibited good computed tomography (CT) and multi-spectral optoacoustic tomography (MSOT) imaging effects in precisely locating and monitoring tumor. Moreover, the antitumor efficacy of GSNP-TPPs + laser group was further confirmed by ex-vivo histological analysis of tumor tissue.

**Conclusion:** This work points out a strategy to overcome the obstacle standing in nanoparticles penetration, and opens the door of further exploitation of NO-related theranostic systems.

## Introduction

Dense extracellular matrix (ECM) of solid tumors presents a delivery barrier that prevents from further nanoparticle penetration, thus limiting the efficacy of cancer theranostics 1. The ECM, mainly composed of hyaluronic acid, collagen and other structural proteins, is one typical feature of tumor microenvironment (TME) 2. Goodman et al. have found the decrease of main matrix component - collagen in ECM can help the particle diffusion in tumor tissues 3. To make the access of nanomedicines to the tumor sites unobstructed, various proteolytic enzymes are firstly considered to disrupt the stromal barriers. Many strategies have been proposed to degrade ECM such as directly intra-tumoral injection of enzymes (eg. hyaluronidase 4 and collagenases 5) and modification of nanocarrier surfaces with enzymes (eg. bromelain 6 and hyaluronidase 7). The protein-based methods, however, may easily lose biological function or shorten blood circulation time *in vivo*. In addition, it is difficult to ensure the stability of enzymes in the blood stream, which may even lead to life-threatening side effects. As a result, it is necessary to design a protein-free system to realize the deep penetration of nanomedicine.

Nitric oxide (NO) is the first typical gaseous molecule that arouses people's interests owing to its good diffusive ability and cell membrane permeability, which possesses wide range of biological activities and therapeutic potentials 8-14. It has been reported that matrix metalloproteinases (MMPs), which contain a family of enzymes that can break down matrix proteins, could be activated by NO for collagen depletion, thus enhancing the penetration of nanoparticles 15,16. What's more, when NO located in tumor site at relatively high concentration, the arisen RNS and superoxide (O_2_Ÿ^-^, which is mainly generated by mitochondria) can ablate tumor cells by inducing oxidative/nitrosative stress, mitochondrial/DNA damage and enhanced inflammatory reactions, thereby causing the acceleration of the cell apoptotic death 17-20. However, there are still concerns about the NO delivery by nanocarriers for the difficulties in high-efficient loading and precise releasing 9,21.

Recent years, researchers have exploited a variety of NO donors 22, 23, such as S-nitrosothiols (SNOs), N-nitrosamines, metal NO complexes, bis-N-nitroso compounds (BNNs), Roussin's black salt (RBS), etc. Thereof, SNOs, the simple organic esters of nitrite and sulfhydryls, exhibit interesting characteristics of the thermal-controlled release, photolytic initiated mechanisms, as well as trans-nitrosation properties 23. Lights as noninvasive, cheap and practicable exogenous stimuli 24 have attracted enormous attention to encourage people to design reasonable SNOs loaded nanocomposites 19. However, most of the photo/thermo-sensitive SNOs loaded nanoparticles are triggered or upregulated by UV light, which is highly phototoxic to skin and poorly penetrable to deep tissue 25. Gold nanoparticles, which are one kind of noble metal materials, have been approved by FDA for clinical trials due to the good biocompatibility and lower toxicity 26. There is a big family existing in gold nanoparticles, including gold nanospheres 27, nanoclusters 28, nanocages 27, nanowires 29, nanorods 27, nanotubes 30, nanostars 31, nanocombs 32, nanobelts 32, nanoshells 33 and so on. Featuring tunable sizes and aspect ratios, gold nanorods exhibit strong localized surface plasmon resonance (LSPR) and sufficient NIR photothermal heat conversion, which are undoubtably demonstrated to be promising candidates in photothermal therapy as well as tumor visualization of optoacoustic (OA) imaging and computed tomography (CT) 34-38. In comparison of larger gold nanorods, mini-sized nanorods (width <10 nm) are superior in photothermal therapy efficiency, cellular uptake, tumor accumulation and organ clearance 39,40. Besides, mesoporous silica modification on the surface endows gold nanorods (GNRs) with excellent biocompatibility 41.

**Figure 1:**
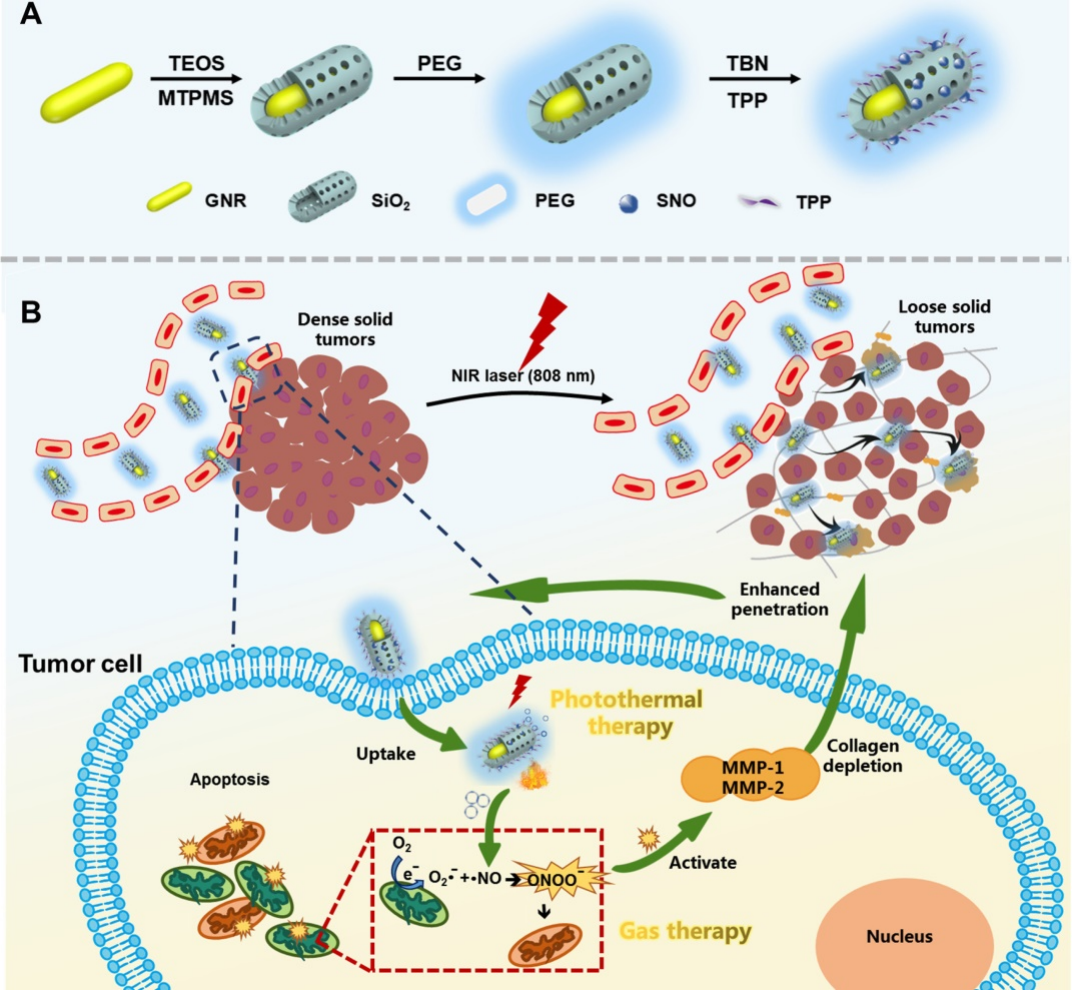
Schematic illustration of A) the synthesis route GSNP-TPPs and B) gas-photothermal therapy.

Herein, we formulated a mitochondrial-targeted protein-free collagen nanosweeper, triphenylphosphonium bromide (TPP, a mitochondria targeting cationic compound) coated and SNOs loaded mini-sized Au@silica nanorod (Au@SiO_2_-SNO/PEG/TPP, GSNP-TPP), which was shown in Figure 1A. During blood circulation (Figure 1B), the nanoparticles were passively accumulated at the tumor sites and efficiently converted light into heat for photothermal therapy (PTT) as well as broke S-NO bond for on-demand NO release upon NIR laser irradiation. On one hand, NO could activate MMPs to transform matrix collagen into a loose state. On the other hand, the diffusible nitric oxide (NOŸ) might target to mitochondria (main source of O_2_Ÿ^-^) and rapidly produce highly toxic peroxide derivative (ONOO^-^) to conversely induce oxidative and nitrification pressure to mitochondria 42. As a consequence, the GSNP-TPPs nanosweeper with low cytotoxicity not only eliminated ECM barriers to enhance the penetration of nanoparticles, but also achieved good antitumor efficiency of photothermal-gas therapy in deep tumor sites as well as CT/MSOT real-time monitor imaging.

## Materials and Methods

### Materials

Cetyltrimethyl ammonium bromide (CTAB) (99% for molecular biology), Annexin V-fluorescein isothiocyanate (FITC)/PI apoptosis detection agents, mitochondrial membrane potential assay kit with JC-1 and 3-(4,5-dimethylthiazol-2-yl)-2,5- diphenyltetrazolium bromide (MTT) were all purchased from Beijing Solarbio Technology Co., Ltd. (China). Hydrogen tetrachloroaurate(III) (HAuCl_4_, 99%), sodium borohydride (NaBH_4_, 97%), 3-(mercaptopropyl) trimethoxysilane (MPTMS, 95%) and tert-butyl nitrite (TBN, 95%) were bought from Tianjin Heowns Biochemical Technology Co., Ltd. (China). Aqueous ammonia (NH_3_▪H_2_O, 28%) was purchased from Shanghai ALADDIN Reagent Co., Ltd. (China). Methanol and ethanol (99.5%) were obtained from Tianjin Yuanli Chemical Co., Ltd. (China). Silver nitrate (AgNO_3_, 99%) was gained from Tianjin Yingda Rare Chemical Reagents Factory (China). Hydroquinone (HQ, 99%) was obtained from Shanghai Rhawn Chemtech Co., Ltd. Tetraethylorthosilicate (TEOS, 28%) was got from Tianjin Damao Chemical Reagent Co., Ltd. (China). (3-Carboxypropyl) triphenylphosphonium bromide (TPP, 97%) was bought from J&K Scientific Ltd. (China). Zinc-phthalocyanine (ZnPc) was obtained from TCI (Shanghai) Development Co., Ltd. Silane-PEG2000-OH was synthesized by Shanghai Pengsheng Biotech Inc. Dulbecco's modified eagle's medium (DMEM, high glucose, GIBCO), fetal bovine serum (FBS, GIBCO) and penicillin/streptomycin were bought from was gained from Invitrogen (USA). Calcein-AM/PI double stain kit was obtained from Shanghai Yeasen Biotech Co., Ltd. (China). NO assay kit and 3-amino,4-aminomethyl-2',7'-difluorescein diacetate (DAF-FM DA) were supplied by Shanghai Beyotime Biotechnology Co., Ltd. (China). Apoptosis was detected by terminal deoxynucleotidyl transferase dUTP nick end labeling (TUNEL) assay with a detection kit (Roche, Switzerland; Cat. No.11684817910). All chemical reagents were of analytical grade and used as received without further purification.

### Preparation of gold nanorods (GNRs)

The gold nanorods were synthesized by a seedless method 43. Firstly, all glassware was washed with *aqua regia* before use. Then, 5 mL HAuCl_4_ (1 mM) was added into 5 mL CTAB solution (0.2 M) to treat with continuous sonication for 1 min. Subsequently, 125 μL AgNO_3_ solution (8 mM) and 1 mL HQ (30 mM) were dropwise added into the previous solution, respectively. Under hand-stirring, the color of the growth solution became colorless within 1 min. Finally, the reaction was started by injecting 15 μL freshly prepared ice-cold NaBH_4_ (10 mM). The mixed solution was slightly shaken until homogeneous and then left in a 30 °C water bath under darkness. After 3 h incubation, the as-synthesized GNRs were obtained by centrifugation (15840 ×g, 15 min) and rinsed once with deionized water to remove excess CTAB surfactant.

### Synthesis of Au@SiO_2_-SH nanorods (GSNs)

The fabrication of the SiO_2_ shells was conducted according to Zihua Wu's procedure 44 with minor modifications. At first, nine equivalent GNR precipitates were collected and re-dispersed in 45 mL of deionized water. The temperature of the solution was kept at 30 °C. Upon stirring, appropriate amount of NH_3_▪H_2_O (28%) was added to adjust the pH value of 10~10.4. Subsequently, three times of 180 µL injections of 10% TEOS in methanol were added to the solution at an interval of 30 min to react overnight. To modify the surfaces of the SiO_2_ shells with -SH groups, 11 μL MTPMS was added and the reaction mixture was transferred to an oil bath with the temperature increasing to 80 °C. After a further 3 h reflux, the Au@SiO_2_-SH nanorods (GSNs) were centrifuged at 13798 ×g for 10 min and washed with water and ethanol several times.

### Synthesis of Au@SiO_2_-SH/PEG (GSP), Au@SiO_2_-SNO/PEG (GSNP) and Au@SiO_2_-SNO/PEG/TPP (GSNP-TPP)

To modify PEG on the nanoparticles, GSNs (4 mg) and silane-PEG2000-OH (~12 mg) were dispersed in 2 mL PEGylation buffer (95% ethanol/5% water, w/w). The mixture was rapidly stirred for 2 h under darkness at room temperature. The resulted PEGylated nanoparticles (GSPs) were obtained by centrifugation (13798 ×g, 6 min) and washed three times with water.

To convert -SH groups to -SNO groups, 4 mg GSP nanoparticles were dissolved in methanol and excess t-butyl nitrite (~180 μL) was dropwise added. The mixture was rapidly stirred for 24 h at room temperature under light-shielded conditions. The resultant S-nitrosothiol-loaded nanoparticles (GSNPs) were centrifuged at 13798 ×g for 5 min, washed three times with anhydrous ethanol, re-dispersed in 4 mL water and stored at 4 °C.

The addition of TPP to the nanocarrier for mitochondrial targeting was reacted through electrostatic interaction. Firstly, 2 mg GSNP nanoparticles and 17 mg TPP were respectively dissolved in 2 mL PBS. Then 500 μL TPP solution was added into GSNP solution to stir for 3 h. The TPP-linked GSNP (GSNP-TPP) was obtained by centrifugation (13798 ×g, 5 min) for 3 times and was freeze dried for test use.

#### Characterization of the nanoparticles

The morphological features and particle size analysis were carried out on transmission electron microscopy (TEM) (JEM-100 CX, Jeol Ltd., Japan). The elemental mapping images were captured by using a high-resolution TEM (JEM-2100f, Japan). The mean particle size and zeta potential of GSNP-TPP were measured by a Zetasizer Nano-ZS (Malvern Instruments, UK). The characteristic spectra were respectively detected by using UV-vis spectrophotometer (Agilent, Santa Clara, USA), FT-IR spectrophotometer (TENSOR 27, Bruker, Germany) and powder X-ray diffractometer (XRD) (D/MAX 2500, Rigaku, Japan). The measurements of nitrogen and phosphorus contents were carried out on ONH elemental analyzer (Leco TC-400, USA) and inductively coupled plasma mass spectrometry (ICP-MS) (Agilent 7700x, USA). The energy-dispersive X-ray spectroscopy (EDS) spectrum was captured by a high-resolution TEM (Thermo Fischer Talos F200x, Czech Republic). Nitrogen physisorption was performed with Quantachrome equipment at 77 K using liquid nitrogen to determine the specific surface area and the pore size distribution by the Brunauer-Emmett-Teller (BET) theory and the Barrett-Joyner-Halenda (BJH) theory, respectively. Thermal images were captured by a 225s infrared camera (Fotric, China) and analyzed by Fotric AnalyzIR image software. The laser light source (808 nm, diode-pumped solid-state laser system, LASERGLOW Technologies, Shanghai, China) was applied to induce photothermal effects.

### Loading measurement of TPP

4.0 mg GSNP-TPPs were accurately weighed and the sample was fully digested by *aqua regia* to form an ionic solution. After the removal of *aqua regia*, we made an aqueous stock solution of the sample (4.0 mg/mL) and diluted it to a ppm level for the following elemental analysis. The loading capacity was calculated according to equation* shown below.

### Photothermal heating experiments

Different concentrations of GSNP-TPP aqueous dispersion (0, 25, 50, 100 and 200 μg/mL) were irradiated with a continuous-wave 808 nm laser at a power density of 1.0 W/cm^2^ for 5 min. The temperatures of the sample dispersion were monitored by the digital thermometer every 30 s, while the photothermal images were taken by an infrared thermal camera. Then, the GSNP-TPP solution (200 μg/mL) was irradiated at a series of power densities (0.8, 1.0 and 1.2 W/cm^2^) for 5 min. For the photothermal conversion efficiency (η), we recorded the temperature change in real time until it tended to stabilize. Then the laser was turned off, and the sample solution was naturally cooling down to the room temperature. Following the formula 45 shown below, the η value was calculated:


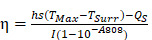
(1)

h is the heat transfer coefficient, S is the surface area of the container, and the value of hS can be gained from the Eq. 4 and Figure 3E. The maximum steady temperature (T_Max_) and environmental temperature (T_Surr_) are 53.6 °C and 23.5 °C, respectively. Q_S_ refers to the heat dissipated from the light absorbed by the container itself (Q_s_= 3.5 mW for pure water). The incident laser power (I) and the absorbance of the GSNP-TPP nanoparticles at 808 nm (A_808_) are 1.0 W/cm^2^ and 1.3229, respectively. A dimensionless parameter θ is calculated as follows:


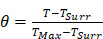
(2)

A sample system time constant τ_s_ can be calculated as Eq. 3.


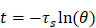
(3)

In terms of Figure 3E, τ_s_ is determined and calculated to be 350.72 s.


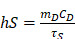
(4)

Additionally, m_D_ is 0.5 g and C_D_ is 4.2 J/g▪ °C. Thus, according to Eq. 4, hS is calculated to be 5.99 mW/°C.

After substituting corresponding values of each parameter to Eq. 1, the photothermal conversion efficiency (η) of the GSNP-TPP nanoparticles could be calculated as 18.56%.

At last, to assess the stability of the as-synthesized nanoparticles, the on/off cycle irradiation experiment was carried out by subjecting the GSNP-TPP solution to 808 nm laser irradiation for 4 cycles.

### *In vitro* and *in vivo* CT and MSOT imaging

For the *in vitro* MSOT imaging of GSNP-TPPs, the solution with various concentrations (0.025, 0.05, 0.2, 0.4, and 2 mg/mL) was prepared and investigated at 808 nm wavelength on a Vevo LAZR PAI system at designed time points and then processed with ImageJ software. Different concentrations of GSNP-TPPs were obtained for *in vitro* CT imaging. The contrast efficiency was quantitatively assessed by the differential contrast in CT values (in HU).

Equation*:


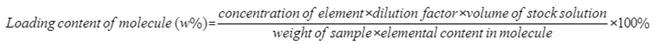


The CT imaging was completed on a micro CT scanner (Quantum FX, PerkinElmer, Hopkinton, MA, USA) with the following operating parameters: 90 kV, 180 µA, 73 mm field of view (FOV), 4.5 min of scan time and Analyze 12.0 software (AnalyzeDirect, Overland Park, KS, USA). Mice were implanted with cancer cells at the right flanks. When the tumor reached an appropriate diameter (~100 mm^3^) and randomly divided into two groups, 100 μL saline and 100 μL GSNP-TPP solution (= 2 mg/mL) were tail vein injected into the two groups, respectively. Commercially used CT contrast agent iopromide (Ultravist® 370 mgI/mL, Bayer, Germany) was applied for comparison. The MSOT and CT images were captured at various time points (0, 2, 6, 12 and 24 h).

### Measurement of the NO release of GSNP-TPP nanoparticles in aqueous solution

The NO release behaviors of GSNP-TPPs were assayed using a Griess reagent kit that could effectively detect the presence of nitrite ions in solution 46. The released NO was easily turned to nitrite and reacted with the Griess agent to form a diazo compound, which showed pinkish color. In this study, two GSNP-TPP solutions (10 mg∙mL^-1^, 2 mL) were respectively kept in the dark and irradiated with 808 nm laser (1 W/cm^2^), and then an aliquot sample (150 µL) was periodically withdrawn at predetermined time points. After that, the 150 µL sample was mixed with Griess reagent and centrifuged (15840 ×g) for 30 min. Then, 150 µL of the supernatant was transferred to a transparent 96-well microplate for test. The signal adsorption at 540 nm was quantified via a microplate reader and the concentration of NO was calculated through a NaNO_2_ standard curve (Figure S1).

### Cell culture

The cancer cell line (HeLa) was purchased from Procell Life Science& Technology Co., Ltd.(Wuhan, China). H9c2 cells were originated from American Type Culture Collection (ATCC) (Manassas, VA, USA) and obtained from the Peking Union Medical College (Beijing, China).

HeLa cells were cultured in DMEM medium (supplemented with 10% FBS, 100 U/mL penicillin and 100 U/mL streptomycin) at 37 ℃ in a 95% humidified atmosphere and 5% CO_2_. H9c2 cells were cultured in DMEM medium (supplemented with 10% FBS) under similar conditions mentioned above.

### Co-localization in the mitochondria

For this study, GSNP-TPPs and GSNPs were labeled with zinc phthalocyanine (ZnPc) (Ex.=676 nm and Em.=681 nm) as described below. Each sample (2 mg) was placed in 5-mL vials and dispersed in 1 mL DMSO. ZnPc DMSO solution (2 mg/mL, 100 μL) was added into the vials respectively and the reaction was kept for 6 h in the dark. After completion of the reaction, the products were separated from mixture by centrifugation (15840 ×g, 10 min) and washed three times to remove unbound ZnPc.

HeLa cells (1×10^6^/ well) were respectively treated with saline, GSNPs (50 μg/mL) and GSNP-TPPs (50 μg/mL) for 6 h in CLSM dishes. After incubation, the cells were washed once with PBS and were cultured with 50 nM MitoTracker Green (15 min) (Ex.=490 nm and Em.=516 nm) at 37 °C in the dark. Once removing the dye, the cells in medium were imaged by confocal microscopy.

### Mitochondrial membrane potential assay

JC-1 (5',6,6'-tetrachloro-1,1',3,3'-tetraethylbenzimidazolylcarbocyanine iodide) is a fluorescent probe which is widely used to detect mitochondrial membrane potential (ΔΨm) by observing fluorescent shifts. When the mitochondrial membrane potential is high, JC-1 aggregates in the matrix of the mitochondria, forming a polymer that exhibits red fluorescence (Ex.=585 nm and Em.=590 nm). When the membrane potential is low, JC-1 stays in monomer state and cannot accumulate in the matrix, thus producing green fluorescence (Ex 515 nm and Em 529 nm). The red/green fluorescence intensity ratio indicates the state of mitochondrial depolarization and the decreased ratio typically refers to the dysfunction of mitochondria 47.

Here, HeLa cells (1×10^6^/ well) were respectively treated for 6 h with saline, 50 µM of GSP-TPPs and GSNP-TPPs in CLSM dishes and were exposed to 808 nm laser (1 W/cm^2^, 5 min). After another 18 h of incubation, the cells were washed once with PBS and cultured with JC-1 working solution for 20 min at 37 °C in the dark. Then JC-1 dye was removed and washed twice with JC-1 buffer. The cells in medium were imaged with a confocal microscope.

### *In vitro* cell viability

We performed the cell viability test via the reduction of the MTT reagent. The HeLa and H9c2 cells were respectively seeded into 96-well plates with a density of 1×10^4^ cells per well and then incubated at 37 °C in 5% CO_2_ for 24 h. Next, HeLa and H9c2 cells were co-incubated with different concentration of GSNP-TPP nanoparticles for 6 h. For NIR-mediated groups, the cells were irradiated with 808 nm laser at a power density of 1 W/cm^2^ for 5 min. After a further 18 h, 100 μL of an MTT dye solution (5 mg/mL in phosphate buffer pH 7.4) was added to each well and incubated for 4 h at 37 °C in 5% CO_2_. The medium was removed, and formazan crystals were solubilized with 150 μL of dimethyl sulfoxide (DMSO). The absorbance of each well was measured by a microplate reader at the wavelength of 490 nm. The dark control experiment was conducted under the identical conditions with the experimental group except for NIR laser irradiation. All experiments were performed in triplicate at least.

### *In vitro* antitumor efficiency studies and cell apoptosis analysis

For antitumor efficiency studies and cell apoptosis analysis, the HeLa cells were firstly seeded in 6-well plates at a density of 1 × 10^6^ cell/well for 24 h. The cells were treated under different conditions: (1) only saline treatment; (2) incubated with GSPs for 24 h; (3) incubated with GSPs for 24 h and irradiated with an 808 nm laser at 1.0 W/cm^2^ for 5 min; (4) incubated with GSNP-TPPs for 24 h; (5) incubated with GSNP-TPPs for 24 h and irradiated with an 808 nm laser for 5 min. The irradiation was employed after 6 h of co-incubation with nanoparticles. After treatment, the live/dead cell staining assay was conducted with calcein-AM/PI double stain kit and imaged with a CLSM system (calcein-AM, Ex 490 nm/Em 515 nm; PI, Ex 535 nm/Em 617 nm). Annexin V-FITC and PI co-staining kit was utilized in apoptosis detection and samples were analyzed by FACScan flow cytometer (BD Biosciences).

### Hemolysis assay

Hemolytic effects of nanoparticles on RBCs were preliminarily evaluated by measuring the absorbance of the released hemoglobin from hemolytic RBCs. A suspension of nanoparticles in PBS was prepared by serial dilution from 200 μg/mL to 12.5 μg/mL. Blood samples were collected from mice in sodium citrate tubes. RBCs were separated from serum by centrifugation (70 ×g, 10 min), then washed three times with PBS solution and diluted 10 folds to prepare RBC suspension. Positive control (+) and negative control (-) was prepared by deionized water and PBS, respectively. Equal volume of RBC suspension and sample solution was mixed and incubated under 37 °C for 2 h. The blood mixture was centrifuged at 10137 ×g for 10 min. Next, the upper supernatant was taken into 96-well plate for absorbance measurement by microplate readers. The hemolysis ratio was calculated according to the formula below:


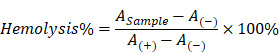


### Western blotting

For Western blot analysis, HeLa cells were seeded in 6-well plates at a density of 2.0×10^6^ cells for 24 h and then treated with varied conditions. The cell pellets were harvested by centrifugation at 70 ×g for 5 min, followed by immerging in lysis buffer and PMSF (Beyotime, China). The total proteins were obtained through centrifugation and processed for Western blot assay using specific antibodies of p53, Bax, Bcl-2, Cleaved Caspase-3, HSP90, MMP-1 and MMP-2, respectively. The test was run three times using the same lysate. The brand, catalog number and the dilution for each antibody used in western blotting were listed in Table S1 (supplemental material).

### Measurement of NO release in HeLa cells

The intracellular NO release was characterized by a NO specific probe, Amino,4-aminomethyl-2',7'-difluorescein, diacetate (DAF-FM DA), in both tumor cells and tissues. DAF-FM DA can penetrate the cell membrane and be catalyzed by intracellular esterase to form membrane-impenetrable DAF-FM. DAF-FM exhibits slight fluorescence, but it can produce benzotriazole (Ex 495 nm, Em 515 nm) with high fluorescence when reacting with NO.

HeLa cells were seeded into CLSM dishes at 5×10^5^ cells/well and then cultured at 37 °C in a humidified standard incubator (5% CO_2_) for 24 h. Later, saline and GSNP-TPPs (50 µg/mL) were respectively added into the dishes and co-incubated for 24 h. Then, the cells were washed with PBS once and loaded with DAF-FM DA probe (5 μΜ) for 20 min. After being exposed to 808 nm laser irradiation, the cells were washed with PBS three times and observed the NO release via CLSM.

### Mouse model and antitumor therapy *in vivo*

Healthy female KM mice (6 weeks) were obtained from Beijing Huafukang Biological Technology Co. The Animal Care and Ethics Committee of the Institute of Radiation Medicine Chinese Academy of Medical Sciences approved all animal experiments for our study (Approval No. IRM-DWLL-2019080), which obeyed the Guide for the Care and Use of Laboratory Animals and the National Institutes of Health's guide for the Care and Use of Laboratory Animals. The right flanks of mice were subcutaneously injected with HeLa cells. When the tumors grew to a volume ~100 mm^3^, the mice were randomly divided into five groups (n = 5).

The mice were respectively administered saline, GSPs (with or without irradiation) and GSNP-TPPs (with or without irradiation) (10 mg∙kg^-1^, 100 μL) through tail veins on day 0, 2, 4, 6 and 8. Next, tumor regions of the laser-treated groups were irradiated with 808 nm laser (1.0 W/cm^2^, 5 min) at 24 h post-injection. The tumor volume, body weight and survival rate of the mice in each group were monitored every other day. The tumors were calculated as volume = (tumor length) × (tumor width)^2^/2. Relative tumor volumes were expressed as V/V_0_ (V_0_ = tumor volume when treatment started). Photographs of mice were also taken using a digital camera. Besides, we examined the histopathology for major organs (liver, spleen, heart, lung, and kidney) and various blood-index monitoring from the control group and treatment group on day 16.

### *In vivo* TUNEL assay

We used TUNEL assay to compare the tumor tissues of control and treatment groups (GSP-TPPs+laser, GSNP-TPPs+laser and GSNP-TPPs+uric acid+laser) of mouse model. We respectively injected saline, GSP-TPPs and GSNP-TPPs in each group into mice with tumors approximately 100 mm^3^ in size. Uric acid (a ONOO^-^ scavenger) was administrated orally at 1.0 g/kg to the mice before the i.v. injection of GSNP-TPPs. The groups treated with GSP-TPPs, GSNP-TPPs and GSNP-TPPs+uric acid were irradiated with 808 nm NIR laser after 24 h. The treatment was carried out every 2 days for 4 times. On day 16, the mice were sacrificed and the tumor tissues were excised. The tumor tissues were detected with a commercial TUNEL kit (Roche, Switzerland; No. 11684817910) according to the manufacturer's protocol.

### Bio-TEM images of cellular uptake

HeLa cells were pre-seeded in 4 mL Dulbecoo's modified Eagle's medium (DMEM) supplemented with 1% penicillin-streptomycin and 10% fetal bovine serum (FBS) at a density of 2.0×10^6^ cells. After 24 h, the growth medium was replaced with fresh DMEM containing 50 μg/mL GSNP-TPPs and then the HeLa cells were respectively incubated for 2 h and 6 h at 37 °C. The cells were harvested by centrifugation at 70 ×g for 5 min. After removing supernatant solution carefully, the cells were fixed by adding 2.5% glutaraldehyde solution. The uptake of nanoparticles within fixed cells were observed by the bio-TEM.

### Statistics analysis

All the experiments were replicated at least three times. Data were denoted as means±standard deviation (SD). The significance of differences was evaluated by Standard Student's t test or ANOVA analysis. The differences were considered statistically significant when p < 0.05 (*), and highly significant when p < 0.01 (**).

## Results and discussion

### Synthesis and characterization of GSNP-TPPs

The preparation process of GSNP-TPPs was shown in Figure 1A. The mini-sized gold nanorods were fabricated according to a seedless method where hydroquinone (HQ) acted as a weak reducing agent in the growth solution for the reduction of Au^3+^ →Au^+^ 48. This method was facile and time-saving, compared with a seed method of gold nanorods synthesis. In the silica-coating process, Au@SiO_2_ nanorods (GSRs) were constructed via the hydrolysis of TEOS. Besides, 3-(mercaptopropyl) trimethoxysilane (MPTMS) was applied for sulfhydrylation of the surface of GSRs before the thiol nitrosation. Then, silane-PEG was conjugated to the silica shell via the reaction between hydroxyl group and ethoxyl/methoxyl silane for the stability improvement of long-term blood circulation. Finally, SNO were covalently linked to the thiolated mSiO_2_ shell by reacting the -SH groups with tert-butyl nitrite (TBN), followed by loading TPP through electrostatic interaction to obtain final structure.

**Figure 2:**
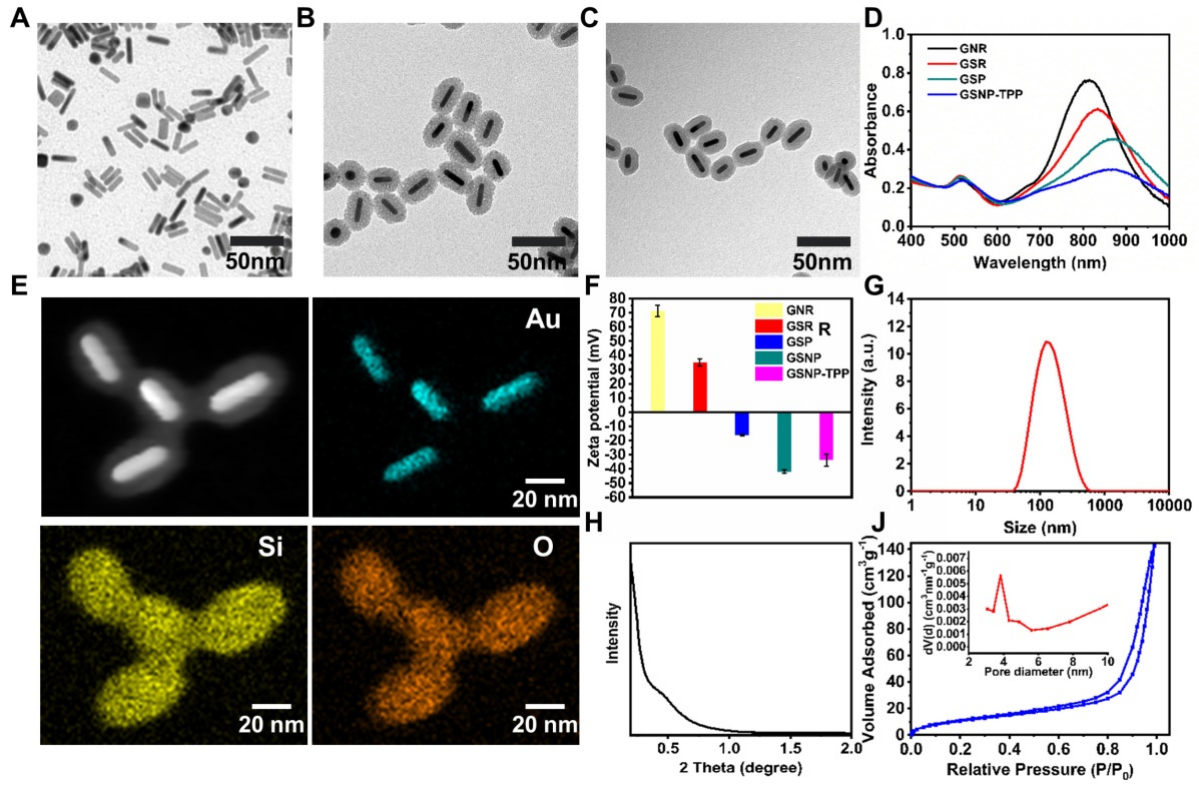
A-C) TEM images of GNR, GSR and GSNP-TPP. D) UV-vis absorption spectrum of GNR, GSR, GSP and GSNP-TPP. E) Energy-dispersive X-ray mapping images of GSNP-TPP. The element maps shows the distribution of Au (blue), Si (yellow) and O (orange). F) Zeta potential of GNR, GSR, GSNP and GSNP-TPP. G) Hydrated particle size of GSNP-TPP. H) Small-angel X-ray diffraction curve of Au@SiO_2_. J) N_2_ adsorption/desorption isotherms and pore size distribution (inset) plots.

The TEM images of GNRs, GSRs and GSNP-TPPs were shown in Figure 2A-C. We obtained mini-sized gold nanorods with average dimensions (length×width) of 32×8 nm with an aspect ratio of ~4. From the TEM image of GSRs and GSNP-TPPs, the mesoporous silica shell was estimated to exhibit a relatively homogeneous thickness of ~13 nm, which was similar to the final framework with small molecule modification. The elemental composition (Au, Si, O) of GSNP-TPPs was confirmed by elemental mapping analysis (Figure 2E), which reflected that GNRs and silicon were encapsulated inside and distributed outside, respectively. Compared with TEM results (32×8 nm), the average hydrodynamic size of GSNP-TPPs measured by DLS was ~110 nm (Figure 2G), which might due to the core-shell structure of the nanorod. In addition, the successful synthesis of each step was observed by the change of zeta potential measurement (from +70 mV to -33 mV) (Figure 2F). Besides, the UV/Vis spectra (Figure 2D) indicated that longitudinal LSPR of the as-prepared GNRs was at 807 nm and exhibited successive red-shifts after each modification. The FT-IR spectra of the GSN, GSP, GSNP and GSNP-TPP from 400 to 4000 cm^-1^ were shown in Figure S2. The absorption peaks at 2922 cm^-1^ and 2850 cm^-1^ caused by the C-H stretching vibration of -CH_3_ and -CH_2_ groups of CTAB were gradually disappeared, indicating that CTAB was removed in the preparation process. The emergence of a new peak of -N=O in GSNP appeared at 1505 cm^-1^, which confirmed the -SNO conjugation 49. However, another characteristic peak of -S-N= at 764 cm^-1^ was not quite obvious due to the low content. The conjunction of TPP was confirmed by the pronounced absorption peak from 1600 to 1400 cm^-1^ in FTIR spectra, which belonged to the three phenyl rings in TPP molecule. Moreover, the successful loading of TPP into nanoparticles was also verified by Zeta potential between GSNP and GSNP-TPP (Figure 2F) as well as Energy-Dispersive X-Ray Spectroscopy (EDS) analysis of P element in GSNP-TPP (Figure S3). GSNP exhibited a zeta potential of -41 mV, while it was enhanced to -33 mV after modification with TPP. From the ICP-MS analysis, the content of P element was measured to 0.15% (w%) in GSNP-TPP, as a result, the loading content of TPP was calculated to be 2.05%. The mapping analysis of S and N elements in Figure S4 confirmed the successful -SNO conjugation. What's more, the content of N element was determined by ONH elemental analyzer (Leco TC-400, USA). The concentration of -SNO was calculated by the percentage of N element in freeze-dried GSNP-TPPs. From the results, the content of N element was 0.19% (w%) in GSNP-TPPs and the concentration of -SNO was calculated to be 8.81 μg/mg. The BET surface area and pore size were measured to be 44.66 m^2^/g and 3.8 nm respectively (Figure 2J). As a result, the BJH pore volume of the core-shell nanoparticles was calculated to be 0.220 m^3^/g, which revealed that the silica layer consisted of mesoporous structure with orderly arranged pores (spectrum of small-angle X-ray diffraction in Figure 2H).

### Photothermal heating experiments and *in vitro* dual-imaging

**Figure 3:**
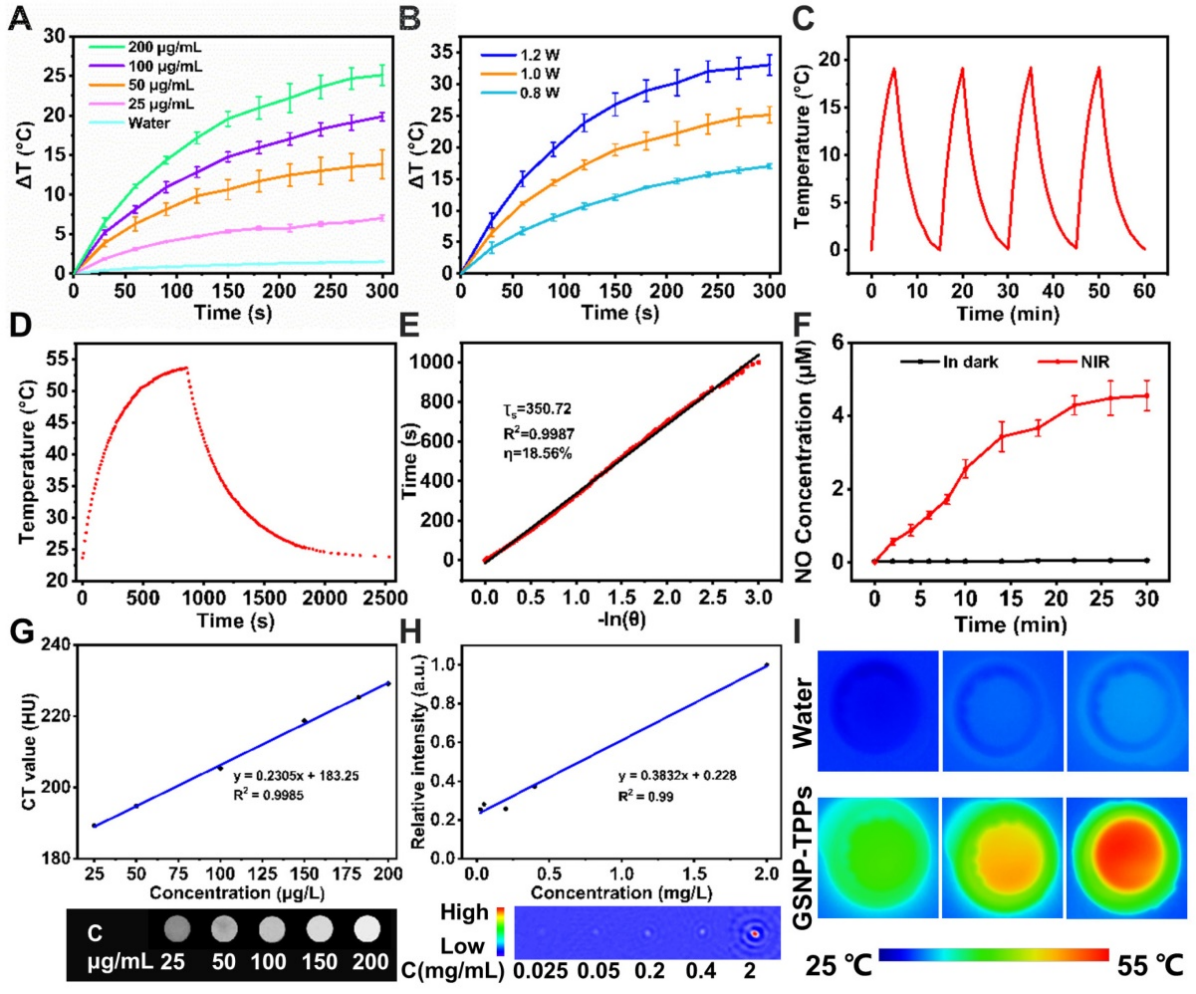
A) The photothermal profiles of pure water and aqueous dispersions of GSNP-TPPs with different concentrations under 808 nm laser irradiation with a power density of 1.0 W/cm^2^. B) Temperature curves of GSNP-TPPs (200 µg/mL) at different power densities (0.8, 1.0, and 1.2 W/cm^2^). C) Temperature elevation curves of GSNP-TPPs (200 µg/mL) over four cycles of 808 nm NIR laser on/off irradiation. D) Photothermal effect of GSNP-TPPs under 808 nm laser (1.0 W/cm^2^). The irradiation lasted for 860 s. E) Plot of cooling time versus negative natural logarithm of the driving force temperature obtained from a cooling stage to calculate time constant (T_s_) for heat transfer. F) NO release profiles of GSNP-TPPs in water with and without continuous (n=3) 808 nm irradiation (1 W/cm^2^). G) *In vitro* HU values of GSNP-TPPs solutions. H) Linear fitting of the MSOT signal intensities as a function of GSNP-TPPs concentration. I) Thermal images of water and GSNP-TPPs aqueous dispersion treated with 808 nm NIR laser irradiation for 5 min.

To achieve proper NIR laser-induced photothermal effects of GSNP-TPPs, gradient concentrations of samples (from 0 to 200 µg/mL) and various power intensities of irradiation (from 0.8 to 1.2 W/cm^2^) were examined by monitoring the elevated temperatures under 808 nm NIR laser irradiation. As a result, the increasing temperature profile of the GSNP-TPPs aqueous dispersion was time and concentration-dependent (Figure 3A). As shown in Figure 3B, 1.0 W/cm^2^ could raise the temperature to more than 50 °C within several minutes, which was sufficient to cause cellular damage. In particular, the increased temperature of 200 µg/mL GSNP-TPPs was 25 °C, which was 16.4 times higher than that of water, implying that the nanoparticles would be effective photothermal agents for cancer therapy. At the same time, real-time thermal images (Figure 3I) were recorded by an infrared thermal camera which visually displayed temperature behaviors. During the four-time cycles of laser on-off irradiation, the GSNP-TPP solution (200 µg/mL) repeatably achieved effective temperature elevation (Figure 3C). To assess the *in vitro* photothermal conversion efficiency, the GSNP-TPPs solution (200 µg/mL) was exposed to a continuous 808 nm laser at a density of 1.0 W/cm^2^ for 860 s (Figure 3D-E). As a result, the obtained data showed that the photothermal conversion efficiency of GSNP-TPPs could be calculated to 18.56%. On the whole, GSNP-TPPs could rapidly convert the energy from 808 nm laser into thermal energy and were qualified to be proper photothermal agents.

Gold nanoparticles (AuNPs) were excellent CT imaging contrast agents due to their high X-ray attenuation, simple surface chemistry, and biocompatibility 20,29. In this study, we tested the *in vitro* CT imaging ability of GSNP-TPPs at various concentrations (= 25, 50, 100, 150 and 200 μg/mL) (Figure 3G). With increasing concentrations, the Hounsfield units (HU) values of CT signals exhibited linearly concentration-dependent increased property.

For *in vitro* MSOT imaging, the photoacoustic signals were produced by gradient concentrations (0.025, 0.05, 0.2, 0.4, and 2 mg/mL) of GSNP-TPPs, which also presented a linear relationship with the increased concentrations (Figure 3H).

### Measurement of the NO release of GSNP-TPP nanoparticles

It is generally assumed that NO release from S-nitrosothiols is caused by homolytic cleavage of the S-N bond at certain conditions 50. Here, the NO release was quantified using a fast and classical Griess assay. As shown in Figure 3F, the maximum concentration of NO could reach to 4.5 μM within 30 min while NO molecules were barely released in the dark, which indicated that NO was able to be rapidly produced from the GSNP-TPPs under NIR radiation.

### Co-localization imaging and mitochondrial membrane potential

To investigate the mitochondrial targeting ability of GSNP-TPPs, saline, ZnPc labeled GSNPs and GSNP-TPPs were respectively incubated with HeLa cells and then were observed via confocal laser scanning microscopy (CLSM). As shown in Figure S5, mitochondria were stained green by MitoTracker Green probe. The red fluorescence was from ZnPc labeled GSNPs and GSNP-TPPs and the overlapping of the co-localization formed a yellow color. The merged images showed that the yellow fluorescence intensity around mitochondria of GSNP-TPPs was the most apparent in comparison of saline and GSNP groups, indicating that the mitochondrial targeting nano-platform GSNP-TPPs had been successfully prepared and selectively delivered to mitochondria.

**Figure 4:**
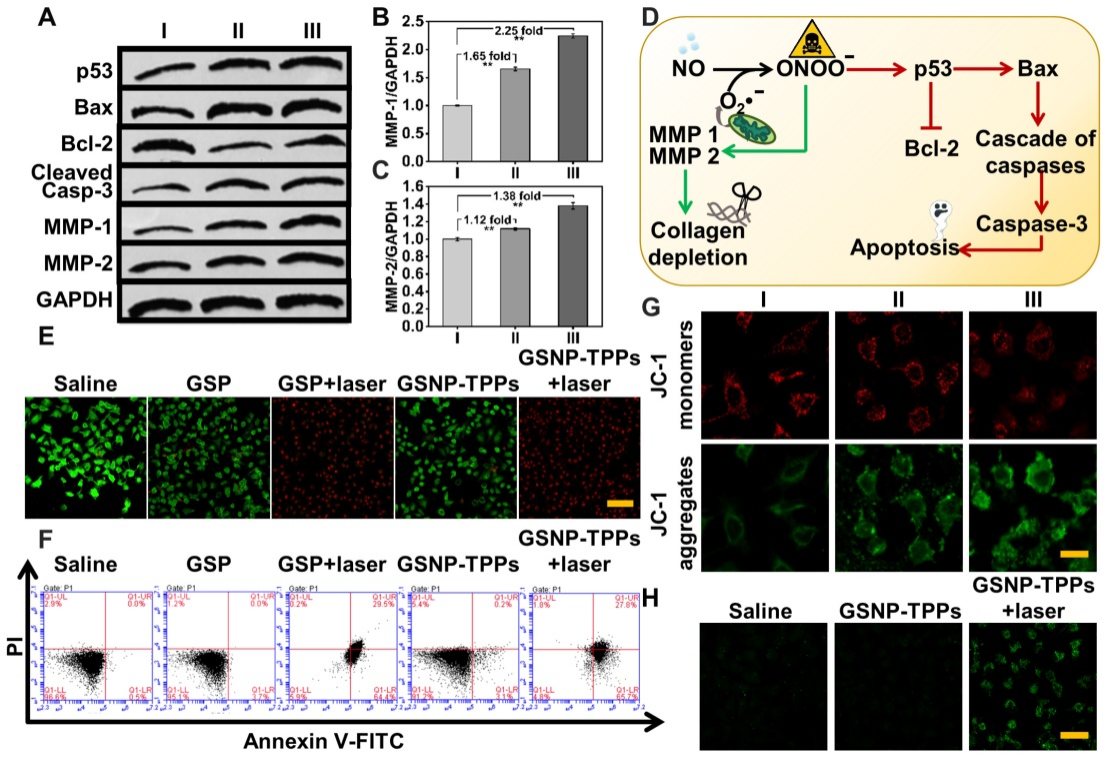
A) Activation of apoptotic signaling pathway and the increased production of MMPs were characterized by western blot under different treatment: I Control, II GSPs+laser, III GSNP-TPPs+laser. B&C) Statistical assay of MMP-1 and -2 contents according to their result of western blot. (n=3, **p < 0.01) D) Schematic illustration of collagen depletion and apoptotic mechanism. E) Fluorescent inverted microscope (scale bars, 100 µm for all panels) of Hela cells stained with calcein AM (live cells, green fluorescence) and PI (dead cells, red fluorescence). F) Flow cytometry assays of HeLa cells treated with saline (control), GSP, GSP + laser, GSNP-TPPs, GSNP-TPPs+ laser. G) Mitochondrial memberane potential (ΔΨm) of HeLa cells after treated with saline (I), GSP-TPPs+laser (II) and GSNP-TPPs+laser (III). Scale bar indicated 25 µm. H) Detection of NO in Hela cells using DAF-FM DA fluorescent probe. Scale bar indicated 50 µm. All the test was carried out in triple.

In this study, JC-1 dye was used as an indicator of changes in mitochondrial membrane potential. When the mitochondrial membrane potential is high, JC-1 aggregates in the matrix as a polymer and generates red fluorescence. On the contrary, JC-1 forms a monomer when the potential is low and produces green fluorescence. The decrease of cell membrane potential is regarded as a sign of mitochondrial damage in terms of the proportional changes of JC-1 by the transition of red to green fluorescence. After HeLa cells were respectively treated with saline (I), GSP-TPPs (II) and GSNP-TPPs (III) for 24 h, the decrease of ΔΨm of GSNP-TPPs treated cells was the most significant, which was verified by the highly increased JC-1 monomer/aggregate ratio (I: II: III=1: 1.38: 2.63) (Figure S6) and the confocal images (the red fluorescence gradually decreased while the green fluorescence enhanced significantly) (Figure 4G), which might be ascribed to the toxicity of ONOO^-^.

### *In vitro* cell viability, antitumor efficiency studies and hemolysis assay

The cytotoxicity of GSNP-TPPs to cells was carried out by means of the standard methyl thiazolyl tetrazolium (MTT) assay and in cell viability assays, GSNP-TPPs showed limited cytotoxicity for both cancerous and normal cells. As depicted in Figure S7, without laser irradiation, the viabilities of GSNP-TPPs treated cells (HeLa, 4T-1 and MCF-7) were all over 90% even if at the highest concentration. For H9c2 cells, less than 13% cells got suppressed with or without light irradiation. However, when the cells were additionally irradiated by 808 nm laser for 5 min at 1 W/cm^2^ power density, their survival rate decreased rapidly with the increasing GSNP-TPPs concentration. Up to 77% of HeLa cells were killed at 200 μg/mL of GSNP-TPPs under NIR light. The CLSM images of live/dead cell staining studies using calcein-AM (green)/PI (red) showed the dominated red color in GSPs+laser and GSNP-TPPs+laser treated group while all the laser-free groups displayed limited cell death, verifying that the laser-plus treatment groups were a potent strategy in killing tumor cells (Figure 4E). In addition, the cell apoptosis was detected by flow cytometry, which displayed that GSNP-TPPs+laser exhibited the highest efficacy (95.3%) to induce apoptosis or necrosis compared to other treatments (group 1: 3.4%, 2: 4.9%, 3: 94.1%, 4: 8.8%) (Figure 4F). Meanwhile, analyzed in concentration range of 12.5 to 200 µg/mL, only about 7.46% hemolysis was observed at concentration as high as 200 µg/mL (Figure S8), showing the good biocompatibility of GSNP-TPPs.

### Western blotting

We further analyzed the expression levels of protein Bax, Bcl-2, p53, Cleaved Caspase-3, MMP-1, MMP-2 and HSP-90 by western blot analysis (Figure 4A and S9). As expected, the anti-apoptotic protein Bcl-2 was suppressed whereas the apoptotic proteins p53, Bax and Cleaved Caspase-3 were upregulated. These proteins were involved in ONOO^-^ caused cascade of caspases in mitochondria and were responsible for apoptosis execution (Figure 4D). In addition, the GSNP-TPPs+laser treatment markedly increased the expression of HSP90, illustrating that the photothermal treatment could induce heat shock pathways in tumor cells.

MMP-1 and -2 are members of interstitial collagenases, which are capable of cleaving fibrillar collagen in normal and malignant cells 51. We detected MMP-1 and -2 expression levels in saline, GSPs+laser and GSNP-TPPs+laser treated groups. As a result, we could find from the western blotting that MMP-1 and -2 were respectively increased by 2.25- and 1.38-fold in NO-releasing nanoparticles treated tumor cells (GSNP-TPPs+laser treated group) compared with the saline group (Figure 4A-C), indicating that the released NO could efficiently activate MMP-1 and -2.

### Measurement of NO release in HeLa cells

The NO generation in HeLa cells was identified by NO probe known as DAF-FM DA. Compared with the rather weak fluorescence in control and GSNP-TPPs group, the cells treated with GSNP-TPPs+laser displayed apparent fluorescence (Figure 4H) and released nearly 2.1-fold increased amount of NO (Figure S10), implying that the GSNP-TPPs were capable of generating NO molecules in live cells upon NIR laser irradiation.

### *In vivo* CT/MSOT dual-imaging and photothermal effects

**Figure 5:**
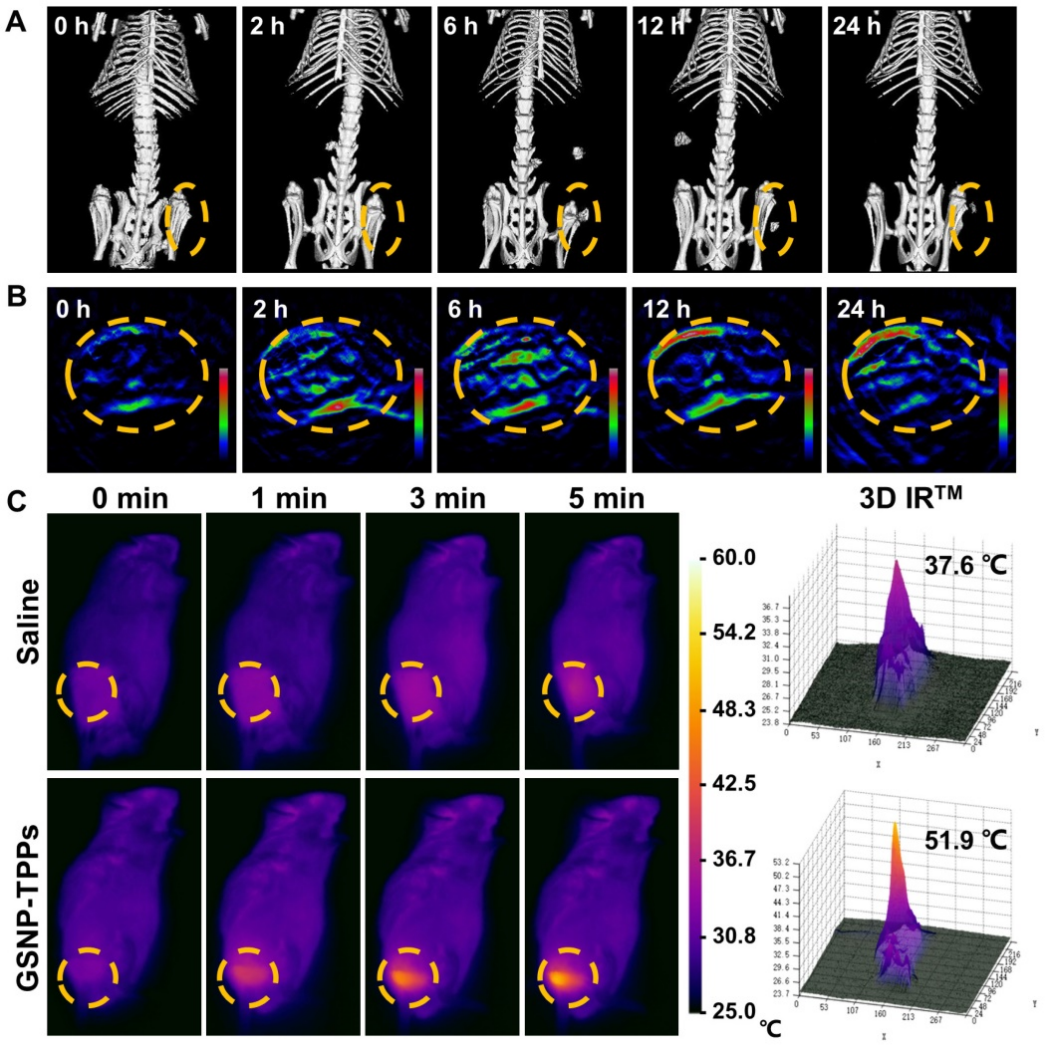
A) *In vivo* CT and B) MSOT images of HeLa tumor-bearing mice after i.v. injection of GSNP-TPPs (2 mg/mL, 100 µL) at different times (0, 2, 6, 12, 24 h). C) Infrared thermographic images of tumor-bearing mice exposed to the NIR laser (808 nm, 1 W/cm^2^, 5 min) after i.v. injection of saline and GSNP-TPPs. Laser was given 24 h post i.v. injection. All highlights indicated the tumor.

To investigate the feasibility of *in vivo* dual-imaging of GSNP-TPPs, we administrated the optimal formulated nanoparticles to tumor-bearing mice. MSOT and CT signals were both recorded at different time points (0, 2, 6, 12, and 24 h), respectively. From Figure 5A and 5B, we could clearly observe that the MSOT and CT signals accumulated in the tumor site and reached a peak at 6 h, indicating the passive targeting ability of GSNP-TPPs owing to the enhanced permeability and retention (EPR) effect. After 24 h, lower intensity signals still could be detected, illustrating the long-time retention of the nanoparticles. Furthermore, we compared the contrast ability of GSNP-TPPs with iopromide through *in vivo* experiments. The CT signal around the tumor was greatly enhanced after i.v. injection of GSNP-TPPs (Figure 5A) and showed much longer blood circulation time (> 6 h), while the commercial CT contrast agent iopromide would be cleaved through kidney within 2 hours. When the CT signal reached to peak, both GSNP-TPPs and iopromide produced clear visualization at the tumor site (Figure S11), which indicated that GSNP-TPPs could be used efficiently as promising CT contrast agents.

We evaluated the photothermal activity of GSNP-TPPs using a thermal imaging camera in HeLa tumor-bearing mice after intravenously injection with saline and GSNP-TPPs, respectively. Then, the tumor sites were exposed to 808 nm laser (1.0 W/cm^2^) irradiation for 5 min, followed by monitoring the temperature changes at specific time intervals (Figure 5C). As expected, the local tumor temperature of the GSNP-TPPs treated mice rapidly increased to 51.9 °C, which was sufficient to eradicate the tumor cells 52-55. However, the tumor surface temperature of the saline treated group displayed a slight change of 4-5 °C during the whole irradiation process. This result indicated that GSNP-TPPs could efficiently convert NIR laser to hyperthermia *in vivo*.

### *In vivo* antitumor efficiency

**Figure 6:**
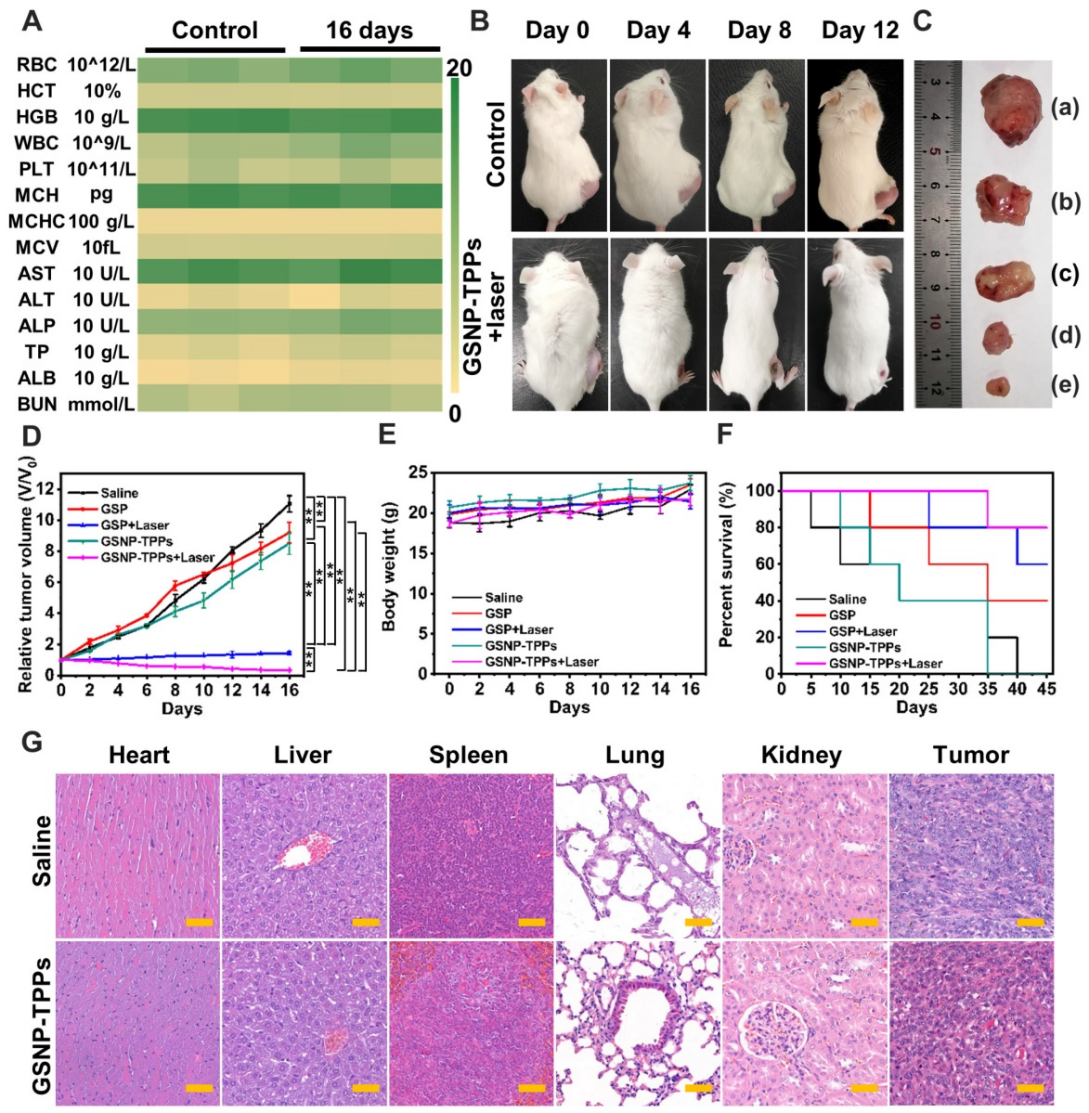
A) Hematological indexes of the mice after 16 days post treatment combined with 808 nm laser (1.0 W/cm^2^). B) Digital images of mice from control and treatment groups in different time intervals. C) Photograph of tumors after excision from each group. (a) untreated, (b) GSPs, (c) GSNP-TPPs, (d) GSPs+laser, (e) GSNP-TPPs+laser. D) Tumor growth and E) weight change curves of tumor-bearing mice after various treatments. F) Survival curves with the various therapeutics (n = 5). G) Histopathological studies of major organs collected from various groups after 16 days post treatment combined with 808 nm laser (1.0 W/cm^2^). These organs are stained with hematoxylin and eosin (H&E) and observed under a light microscope. Scale bar indicated 50 µm.

The photo-responsive antitumor efficacy based on GSNP-TPPs were next evaluated in animal experiments. We tested various blood-index of saline and GSNP-TPPs treated mice, including red blood cell (RBC), hematocrit (HCT), hemoglobin (HGB), white blood cell (WBC), platelets (PLT), mean corpuscular hemoglobin (MCH), mean corpuscular hemoglobin concentration (MCHC), mean corpuscular volume (MCV), aspartate transaminase (AST), alanine aminotransferase (ALT), alkaline phosphatase (ALP), total protein (TP), platelets (ALB) and blood urea nitrogen (BUN). Among them, there were no significant fluctuations in the hematological parameters of the treated group when compared with the control group (Figure 6A). The observation suggested that GSNP-TPPs induced no serious hepatotoxicity and nephrotoxicity *in vivo*.

For the antitumor efficiency experiments, the tumor-bearing mice were randomly divided into five groups: (a) saline, (b) GSPs, (c) GSPs + laser, (d) GSNP-TPPs, (e) GSNP-TPPs + laser. During treatment period, the digital images, tumor growth and body weight were continuously monitored every 2 days. In Figure 6B, apparent shrinkage of the tumor sizes was observed in GSNP-TPPs + laser group compared with the control group during the feeding period. And the tumor volume as a direct index to rate antitumor effects also showed that the tumor growth was suppressed efficiently in the GSPs+laser and GSNP-TPPs+laser groups (Figure 6C-D). More importantly, the optimal treatment (GSNP-TPPs+laser) could reduce the tumor weight significantly (p < 0.01) with the mean tumor weight of 32.8±18.0 mg compared to the untreated tumor-bearing group (997.4±175.9 mg) (Figure S12). Meanwhile, we found that there was no significant difference in the body weight (between 18.6 and 23.7 g), which meant GSNP-TPPs had relatively low systemic side effects during treatment (Figure 6E). In contrast with the laser groups, the tumor inhibition effects were not obvious in solo GSPs or GSNP-TPPs treated group. What's more, GSNP-TPPs+laser achieved the optimal percent survival rate of 80% after 16 days, while mice of other treated groups performed lower survival percentage because of the extensive tumor burden (Figure 6F). Then the pathological changes were further investigated through hematoxyline-eosin (H&E) staining (Figure 6G). By comparison with the control group (saline treatment), there were no apparent signs of inflammatory response, cell degeneration, or necrosis in any of the major organs. In the treatment group, however, obvious tumor damages could be found in the tumor site including cell shrinkage and nucleus loss. These results were in accord with the weight changes and blood chemistry analysis, which demonstrated the biosafety of GSNP-TPPs.

### *In vivo* TUNEL assay and Bio-TEM images of cellular uptake

**Figure 7:**
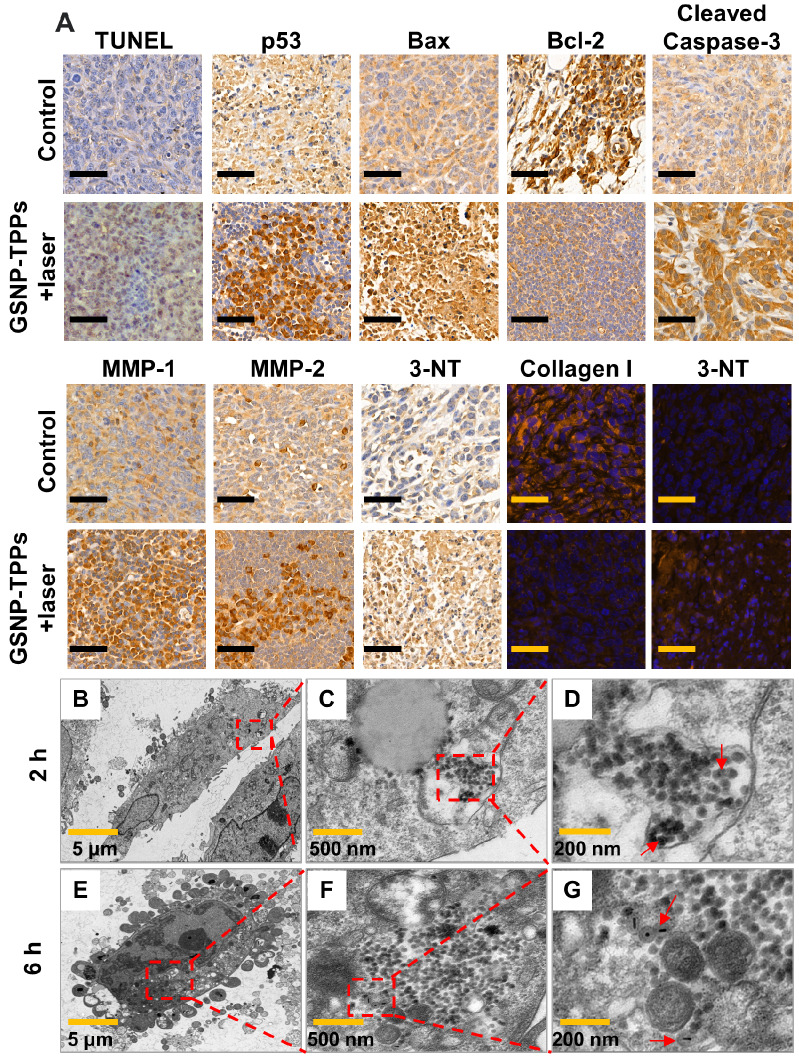
A) TUNEL assay, immunohistochemical and immunofluorescent staining of tumor sections from control and treatment groups. Immunohistochemical staining for p53, Bax, Bcl-2, Cleaved Caspase-3, MMP-1, MMP-2 and 3-NT proteins. Immunofluorescent staining for Collagen I and 3-NT. Scale bar indicated 50 µm. B-G) Bio-TEM images of HeLa cells incubated with GSNP-TPPs after 2 and 6 h.

We performed terminal deoxynucleotidyl transferase dUTP nick end labeling (TUNEL) assays to further test the apoptosis pathway of the tumors. As shown in Figure 7A, S13 and S14, large amount of TUNEL-positive cells were observed in treatment groups (GSP-TPPs+laser 54.72±4.51%, GSNP-TPPs+laser 58.83±4.27% and GSNP-TPPs+uric acid+laser 50.12±3.15%), compared with minimal TUNEL-positive cells in control group (1.25±0.22%), which indicated that a considerable number of HeLa cells were killed through apoptosis by the hyperthermia and NO combined therapy. In addition, the qualitative immunohistochemistry results for p53, Bax, Bcl-2, Cleaved Caspase-3, MMP-1, MMP-2 and HSP90 protein expression were consistent with western blotting measurement (Figure 7A, S13 and S15). The decreased Ki67 (a proliferation marker) expression reflected apparent tumor proliferation suppression of GSNP-TPPs+laser treatment.

In this study, a biomarker 3-nitrotyrosine (3-NT) was used to detect the ONOO^-^ activity in proteins, which was the first step for MMP production. Immunohistochemical and immunofluorescent staining against 3-NT were conducted at 48 h after the final treatment. Compared to saline and GSP-TPPs injection, the increased 3-NT amount (Figure 7A) demonstrated that the administration of GSNP-TPPs enhanced ONOO^-^ levels in treated tumors. In addition, uric acid (a ONOO^-^ scavenger) was utilized to suppress the 3-NT formation and further block the subsequent MMP activation. From the results (Figure 7A and S13), we could observe that the GSNP-TPPs+laser treated group showed the obviously increased MMP expression compared with GSNP-TPPs+uric acid+laser treated group. On behalf of the main matrix component, we chose collagen I as the study object to evaluate depletion effects. When MMP levels declined, the collagen levels increased, indicating the ability of MMP in breaking collagen fibers. With the amount of ONOO^-^ decreased (due to the block of scavenger uric acid), the 3-NT formation was lower while the levels of relative proteins (MMP-1&-2) and collagen I changed slightly. Taking the optimal treatment group into consideration, we could clearly observe that MMP-1&-2 increased and collagen I was downregulated in the GSNP-TPPs+laser treated tumors following the formation of ONOO^-^. In terms of the results of western blotting, TUNEL assay and immunohistochemistry (including p53, Bax, Bcl-2, Cleaved Caspase-3, Ki67), we summarized a scheme of collagen depletion and apoptotic mechanism (Figure 4D), illustrating that the photothermal therapy and ONOO^-^ toxicity were able to induce the classic apoptotic pathway to cause cascade of caspases in mitochondria and apoptosis execution.

Visual proof for the cellular uptake of GSNP-TPPs into HeLa cells was presented through bio-TEM. From Figure 7B-G, we could see that GSNP-TPPs were internalized into the HeLa cells after 2 h and maintained their morphological properties (shape and size) even at 6 h, which could contribute to the photothermal activity and highly efficient damage to tumor cells.

## Conclusion

We successfully fabricate a protein-free collagen nanosweeper by facile assembly of mini-sized Au@SiO_2_ nanorods with SNO and TPP for dual-imaging guided photo-responsive tumor treatment. In this intelligent nanoplatform, the NIR induced hyperthermia leads to NO release for loosening ECM of solid tumors by virtue of NO activated MMP-1 and-2, instead of directly attaching collagenase to nanovehicles. Moreover, the *in vitro* and *in vivo* experiments show that the enhanced penetration and accumulation facilitate nanoparticles to the deep tumor site, thus inducing mitochondrial damage due to the photothermal effects and toxic NO derivative (ONOO^-^). Meanwhile, the potential of GSNP-TPPs as a dual-imaging tool has been proven by recording strong CT and MSOT signals in the tumor areas, which could be demonstrated to conveniently monitor the biodistribution of nanoparticles as well as their antitumor efficacy. This work encourages a further exploitation of NO-related theranostic systems for solid tumor therapy.

## Supplementary Material

Supplementary figures and tables.
