# Supplementary material for: S-nitrosothiols loaded mini-sized Au@silica nanorod elicits collagen depletion and mitochondrial damage in solid tumor treatment
Source: Theranostics. 2020 May 20;10(15):6774–89. doi: 10.7150/thno.42661 (PMC7295055; doi:10.7150/thno.42661)
Supplement: Supplementary file 1 — Supplementary figures and tables. [file thnov10p6774s1.pdf]

Supporting Information

S-nitrosothiols loaded mini-sized Au@silica nanorod elicits collagen depletion and mitochondrial damage in solid tumor treatment

Ping Liu[1], Yidan Wang[1], Yang Liu[3], Fengping Tan[1], Jining Li[2]✉ and Nan Li[1]✉

1. Tianjin Key Laboratory of Drug Delivery & High-Efficiency, School of Pharmaceutical Science and Technology, Tianjin University, 300072, Tianjin, P. R. China.
2. School of Precision Instrument and Opto-Electronics Engineering, Tianjin University, 300072, Tianjin, P. R. China.
3. Department of Orthopedic Surgery, Tianjin Medical University General Hospital, 300052, Tianjin, P. R. China

✉ Corresponding authors: Email: linan19850115@163.com (N. Li); jiningli@tju.edu.cn (J. Li).

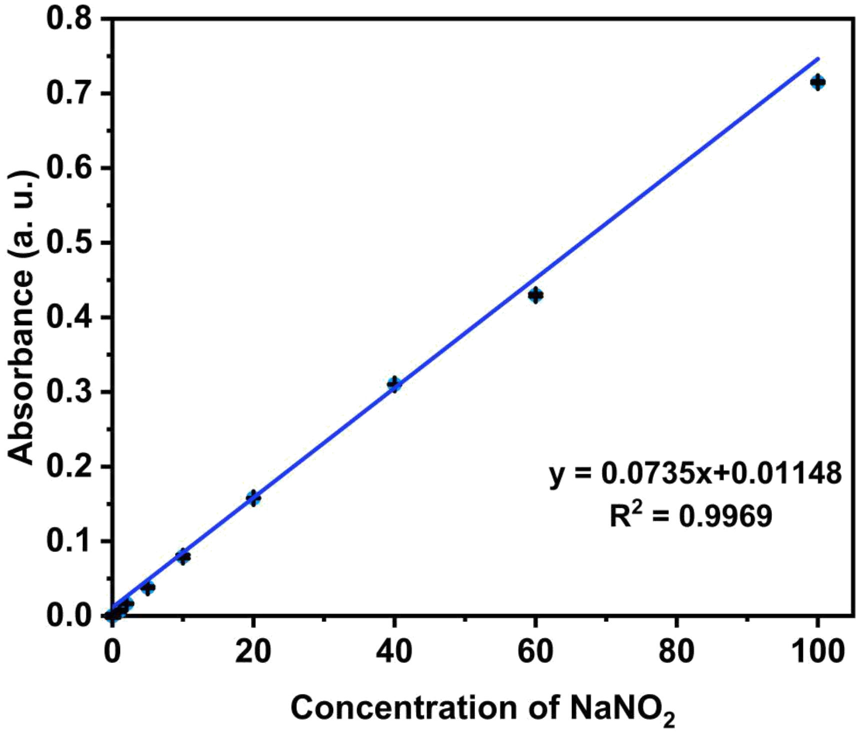

Figure S1. Standard curve of $NaNO_2$ standard samples (0-100 μM).

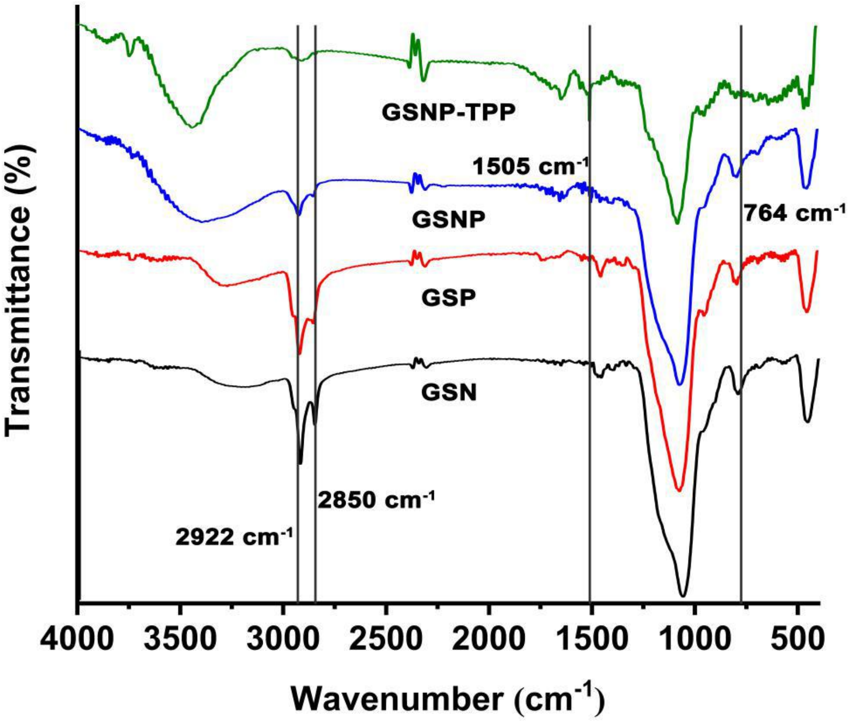

Figure S2. FTIR spectra of GSN (black), GSP (red), GSNP (blue) and GSNP-TPP (green).

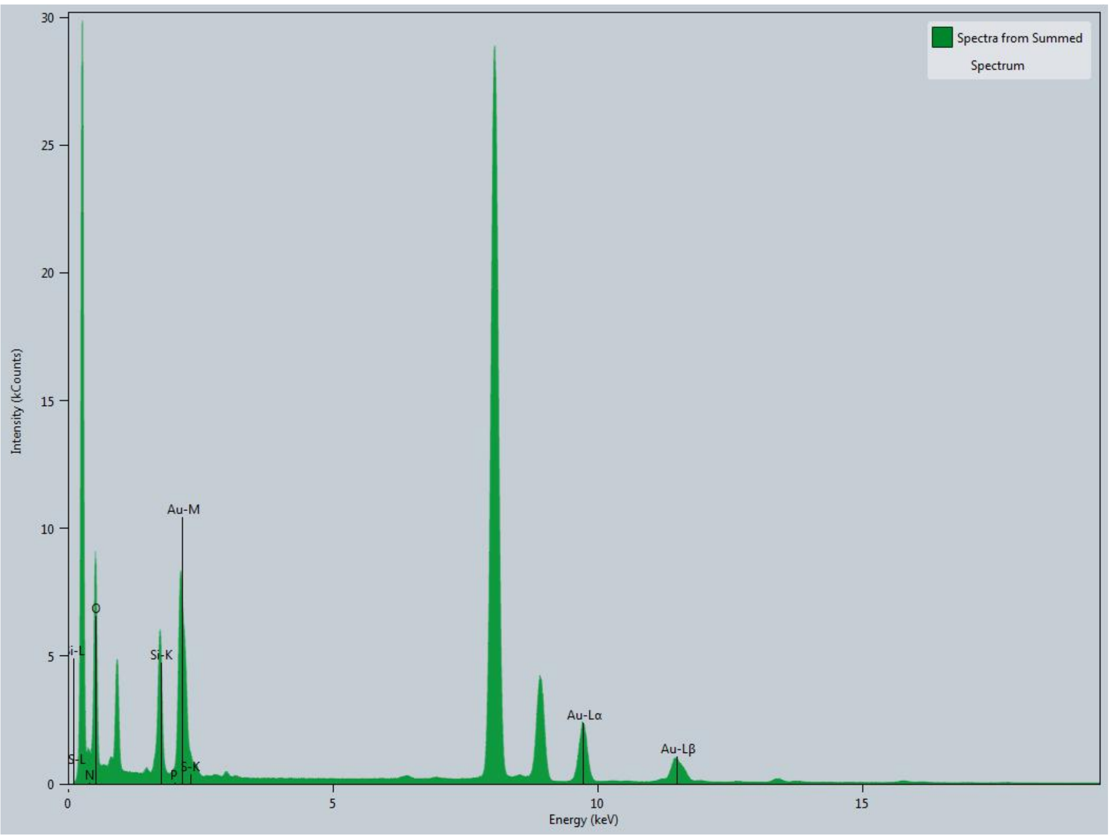

Figure S3. EDS spectra of GSNP-TPP.

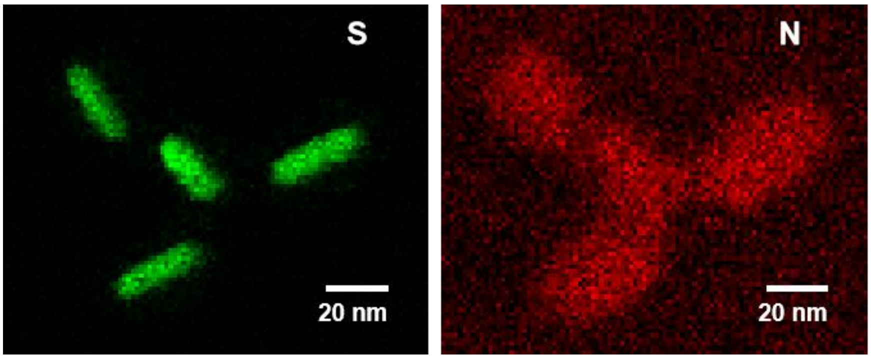

Figure S4. Elemental mapping analysis of S and N in GSNP-TPP.

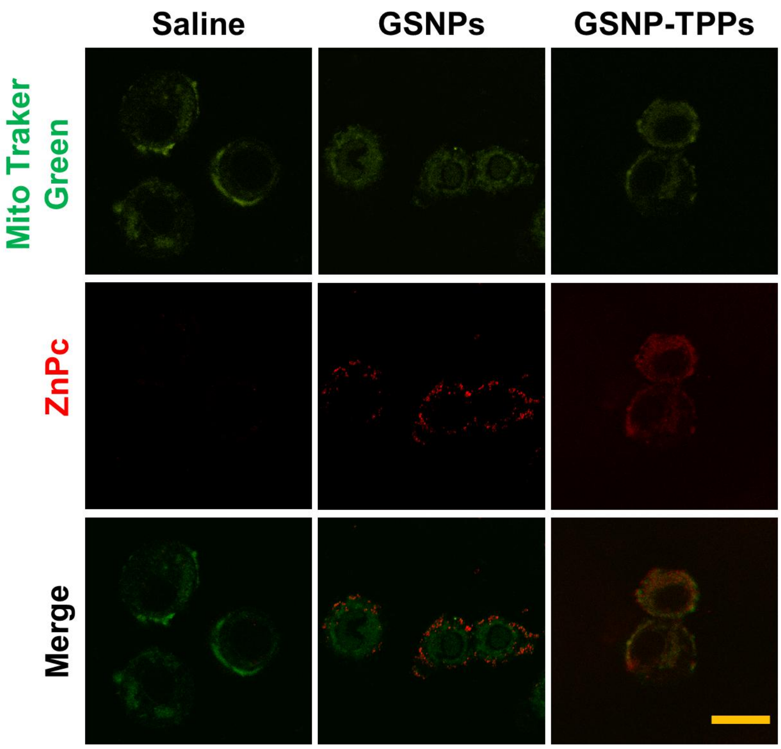

Figure S5. Confocal microscopy images of HeLa cells treated with saline, GSNPs and GSNP-TPPs. GSNPs and GSNP-TPPs were labeled with ZnPc and emitted red fluorescence. Mitochondria were stained by 50 nM Mito-Tracker Green and emitted green fluorescence. Scale bar indicated 25 μm.

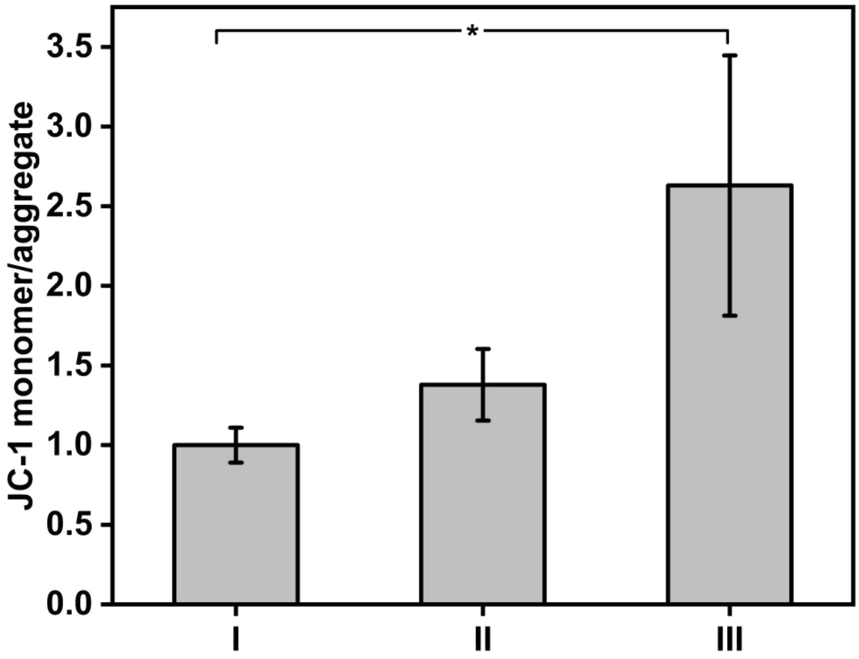

Figure S6. Statistical assay of JC-1 monomer/aggregate ratio shown in Figure 4G. I, II, III were indicated saline, GSP-TPPs+laser and GSNP-TPPs+laser, respectively. (n=3, *p < 0.05)

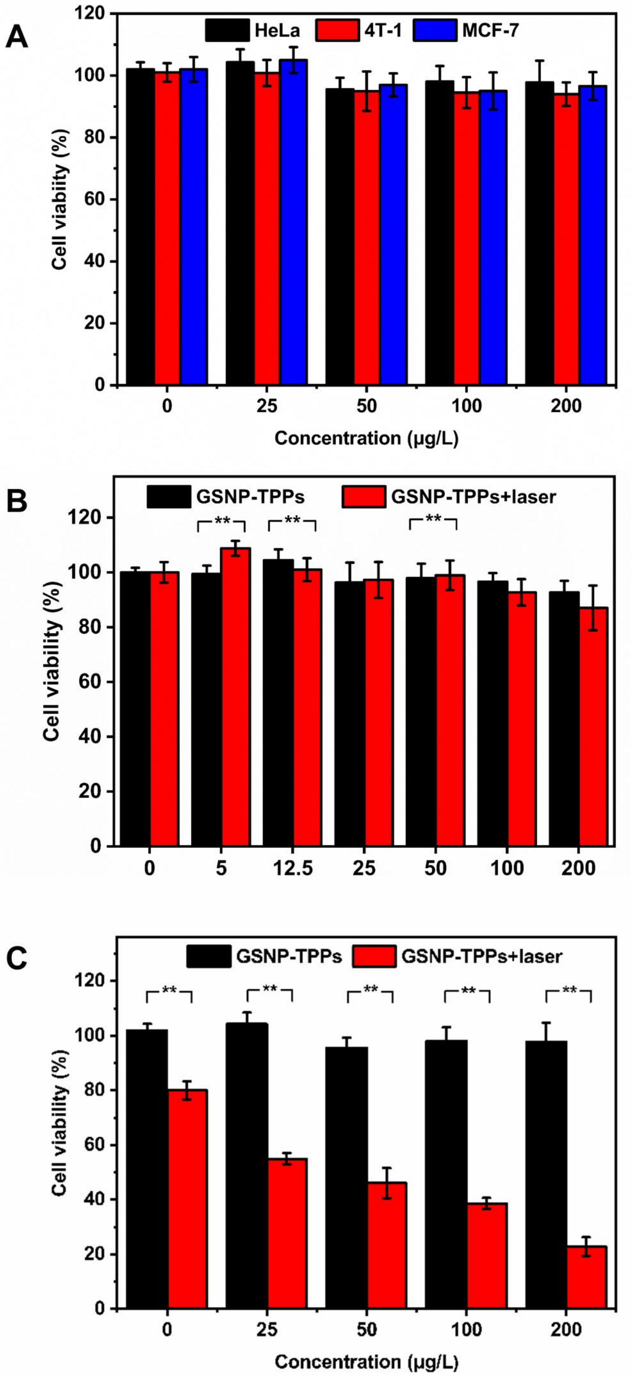

Figure S7. A) Cell viability of HeLa, 4T-1 and MCF-7 cells after treatment with various concentrations (0, 25, 50, 100 and 200 μg/mL). B) Cell viability of H9c2 cells after treatment with various concentrations (0, 5, 12.5, 25, 50, 100 and 200 μg/mL). C) Cell viability of HeLa cells after treatment with various

concentrations (0, 25, 50, 100 and 200 μg/mL). ($n=3$, $^{**}p < 0.01$)

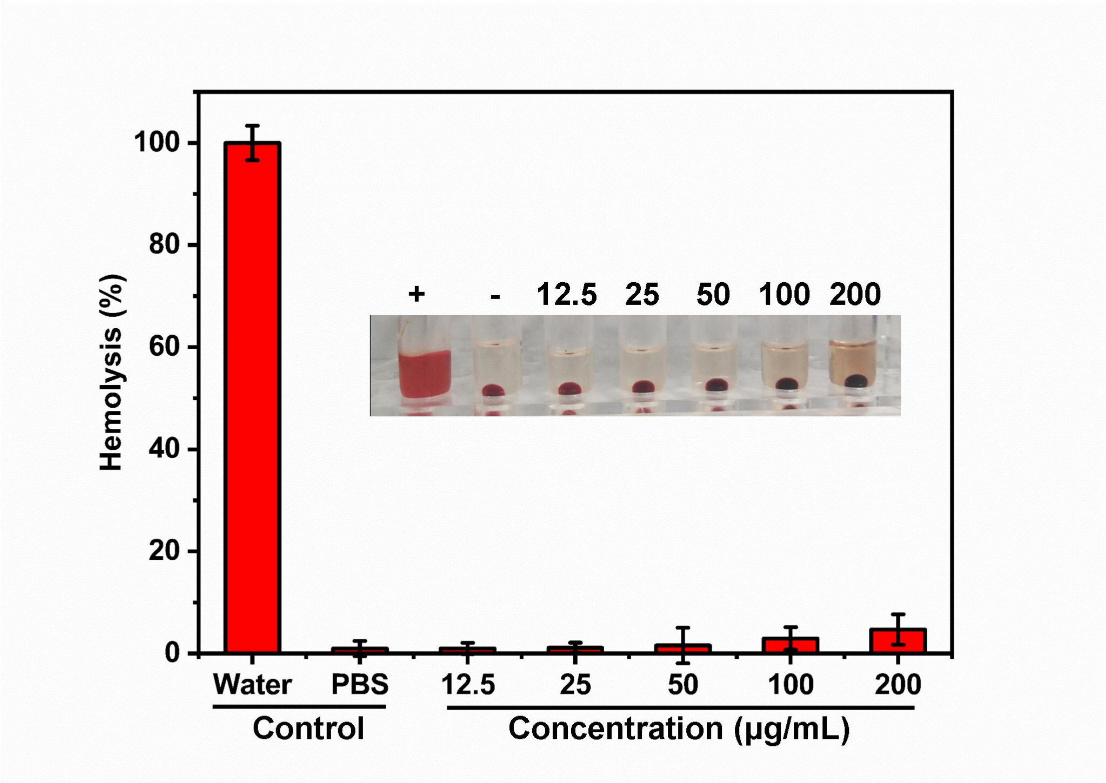

Figure S8. In vitro hemolysis assay of GSNP-TPPs. Inset: hemolysis photos after centrifugation.

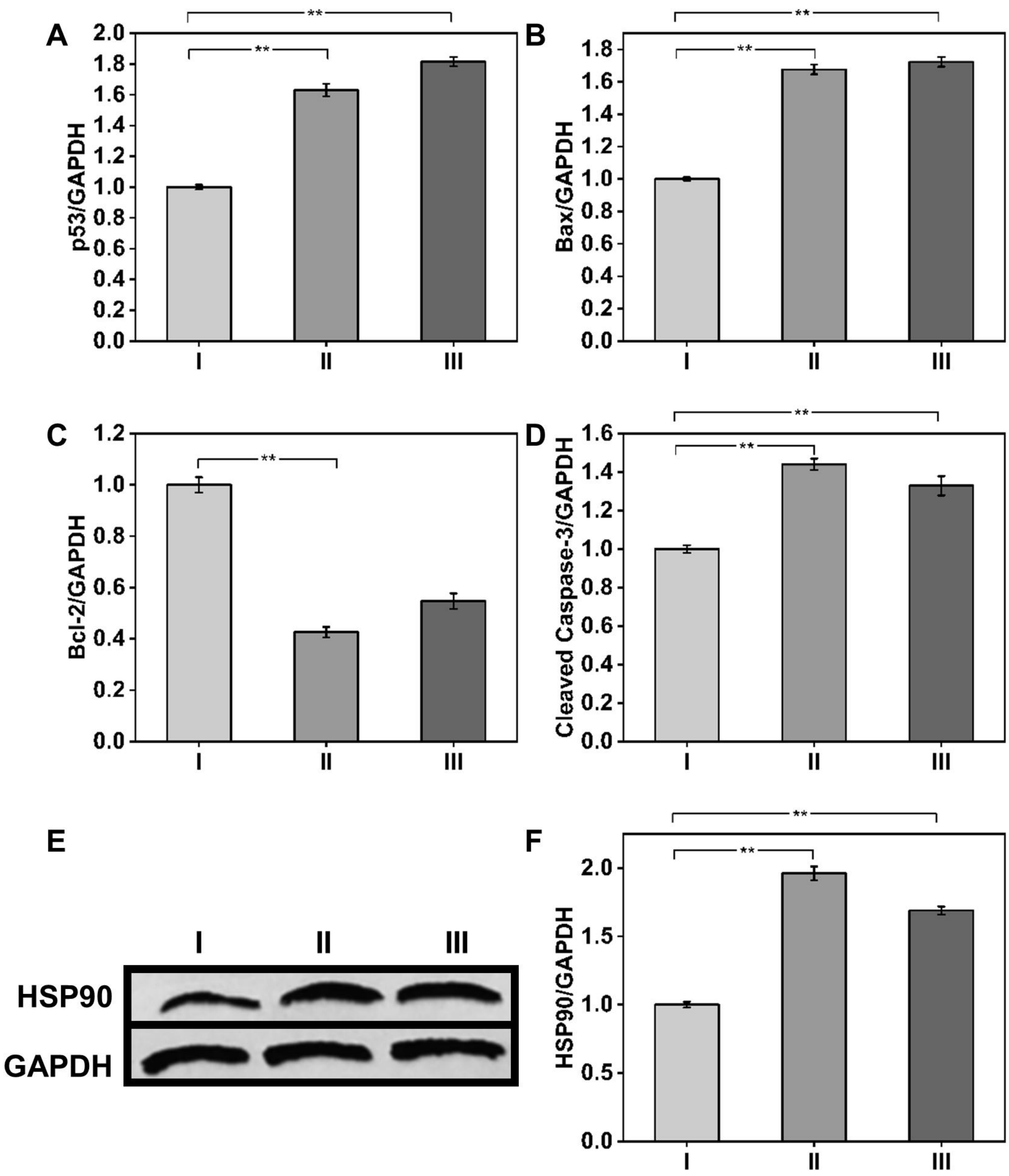

Figure S9. A-D, F) Statistical assay of p53, Bax, Bcl-2, Cleaved Caspase-3 and HSP90 contents according to their result of western blot. (n=3, **p < 0.01) E) Western blot of HSP90 with different treatment: I Control, II GSPs+laser, III GSNP-TPPs+laser.

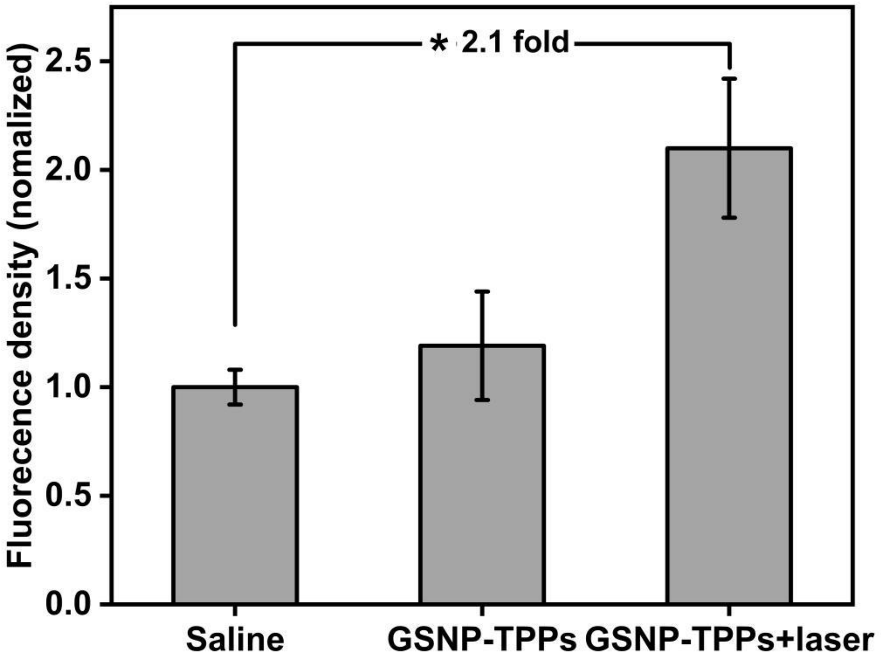

Figure S10. Quantitative comparison of the green fluorescence intensity shown in Figure 4H. (n=3, *$p < 0.05$)

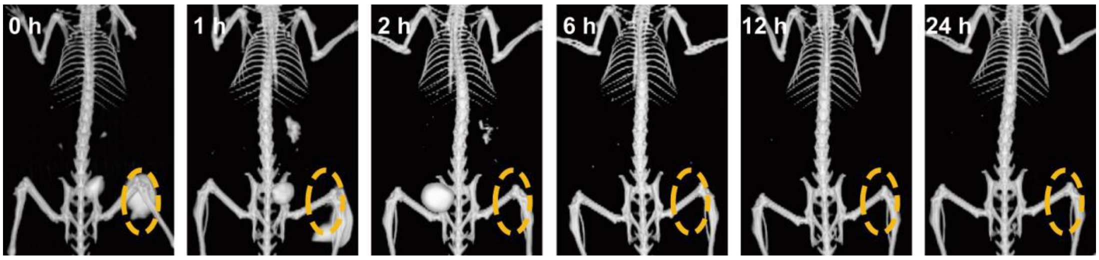

Figure S11. In vivo CT images of HeLa tumor-bearing mice after i.v. injection of iopromide solution at different times (0, 1, 2, 6, 12, 24 h).

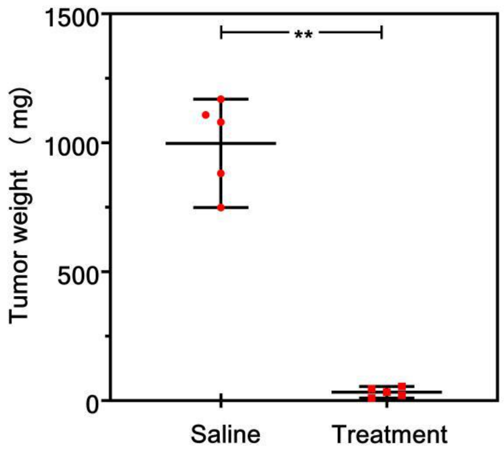

Figure S12. Represents the tumor weights in control (saline) and treatment (GSNP-TPPs+laser) groups after removal of the tumor from animals. Each point represents the weight of each tumor and the lines represent the mean value. “*” denotes statistical difference (**p < 0.01).

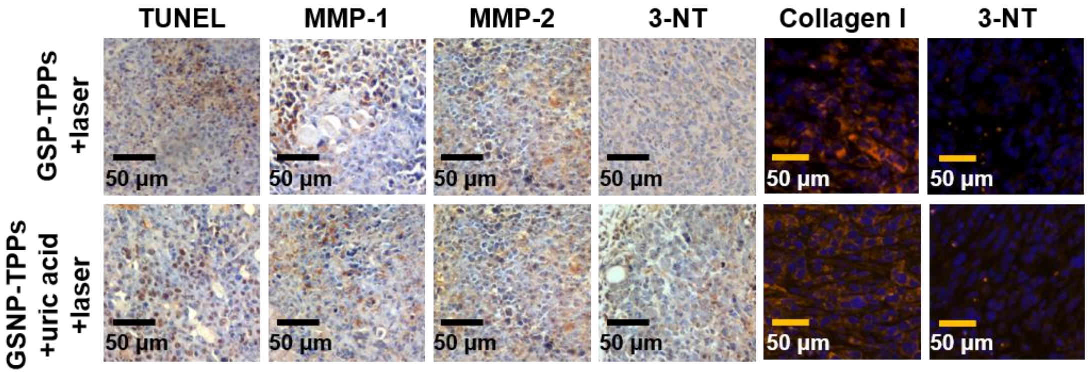

Figure S13. TUNEL assay, immunohistochemical and immunofluorescent staining of tumor sections from GSP-TPPs+laser and GSNP-TPPs+uric acid+laser groups. Immunohistochemical staining for MMP-1, MMP-2 and 3-NT proteins. Immunofluorescent staining for Collagen I and 3-NT.

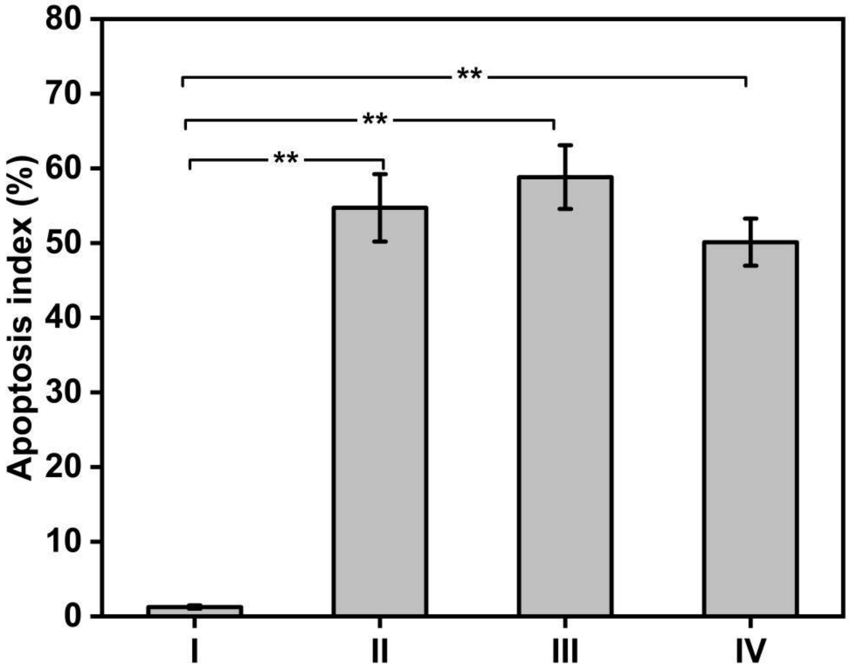

Figure S14. Quantification of the percentage of apoptotic cells in the TUNEL assay in Figure 7 and S13. I Control, II GSP-TPPs+laser, III GSNP-TPPs+laser, IV GSNP-TPPs+uric acid+laser. (n=5, **$p < 0.01$)

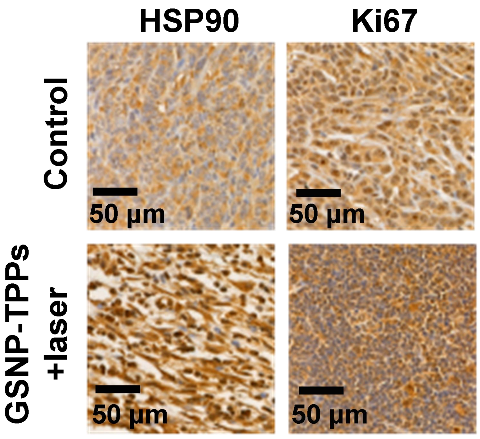

Figure S15. Immunohistochemical staining of tumor tissues for HSP90 and Ki67 proteins.

Table S1. The brand, catalog number and the dilution for each antibody used in western blotting

Antibody	Brand	Catalog number	Dilution
p53	Solarbio	K101293P	1：500
Bcl-2	Solarbio	K003505P	1：1000
Bax	Solarbio	K001593P	1：1200
Cleaved Caspase-3	SAB	29034	1：1200
HSP90	Solarbio	K106929P	1：500
MMP-1	Solarbio	K000342P	1：500
MMP-2	Solarbio	K101302P	1：500
GAPDH	Solarbio	K200057M	1：1000
